# Utility of social media and crowd-intelligence data for pharmacovigilance: a scoping review

**DOI:** 10.1186/s12911-018-0621-y

**Published:** 2018-06-14

**Authors:** Andrea C. Tricco, Wasifa Zarin, Erin Lillie, Serena Jeblee, Rachel Warren, Paul A. Khan, Reid Robson, Ba’ Pham, Graeme Hirst, Sharon E. Straus

**Affiliations:** 1 Li Ka Shing Knowledge Institute of St. Michael’s Hospital, 209 Victoria Street, East Building, Toronto, ON M5B 1W8 Canada; 2 Epidemiology Division, Dalla Lana School of Public Health, University of Toronto, 6th Floor, 155 College St, Toronto, ON M5T 3M7 Canada; 3 Department of Computer Science, University of Toronto, 10 King’s College Road, Toronto, ON M5S 3G4 Canada; 4 Department of Geriatric Medicine, Faculty of Medicine, University of Toronto, 27 Kings College Circle, Toronto, ON M5S 1A1 Canada

**Keywords:** Surveillance, Adverse event, Drug safety, Social media, Data analytics, Knowledge synthesis

## Abstract

**Background:**

A scoping review to characterize the literature on the use of conversations in social media as a potential source of data for detecting adverse events (AEs) related to health products.

**Methods:**

Our specific research questions were (1) What social media listening platforms exist to detect adverse events related to health products, and what are their capabilities and characteristics? (2) What is the validity and reliability of data from social media for detecting these adverse events? MEDLINE, EMBASE, Cochrane Library, and relevant websites were searched from inception to May 2016. Any type of document (e.g., manuscripts, reports) that described the use of social media data for detecting health product AEs was included. Two reviewers independently screened citations and full-texts, and one reviewer and one verifier performed data abstraction. Descriptive synthesis was conducted.

**Results:**

After screening 3631 citations and 321 full-texts, 70 unique documents with 7 companion reports available from 2001 to 2016 were included. Forty-six documents (66%) described an automated or semi-automated information extraction system to detect health product AEs from social media conversations (in the developmental phase). Seven pre-existing information extraction systems to mine social media data were identified in eight documents. Nineteen documents compared AEs reported in social media data with validated data and found consistent AE discovery in all except two documents. None of the documents reported the validity and reliability of the overall system, but some reported on the performance of individual steps in processing the data. The validity and reliability results were found for the following steps in the data processing pipeline: data de-identification (*n* = 1), concept identification (*n* = 3), concept normalization (*n* = 2), and relation extraction (*n* = 8). The methods varied widely, and some approaches yielded better results than others.

**Conclusions:**

Our results suggest that the use of social media conversations for pharmacovigilance is in its infancy. Although social media data has the potential to supplement data from regulatory agency databases; is able to capture less frequently reported AEs; and can identify AEs earlier than official alerts or regulatory changes, the utility and validity of the data source remains under-studied.

**Trial registration:**

Open Science Framework (https://osf.io/kv9hu/).

**Electronic supplementary material:**

The online version of this article (10.1186/s12911-018-0621-y) contains supplementary material, which is available to authorized users.

## Background

Each year, thousands of people die from an *adverse drug reaction*, defined as an undesirable health effect that occurs when medication is used as prescribed [1]. Adverse drug reactions can vary from a simple rash to more severe effects, such as heart failure, acute liver injury, arrhythmias, and even death [1]. These events have a significant impact on both patients and the health care system in terms of cost and health service utilization (e.g., frequent visits to physicians and emergency departments, hospitalizations) [2].

Post-marketing adverse drug reaction surveillance in most countries is suboptimal and consists largely of spontaneous reporting. It is estimated that spontaneous reporting systems only capture 1–10% of all adverse drug reactions. For example, one out of every five physicians reports an adverse drug reaction using the Canada Vigilance Database [3].

In order to advance *pharmacovigilance* (defined as the science and activities related to detection, comprehension and prevention of adverse drug events) [4], monitoring and analysis of data collected from social media sources (i.e., social media listening) is being researched as a potential to supplement traditional drug safety surveillance systems. Three reviews [5–7] have been recently published to explore the breadth of evidence on the methods and use of social media data for pharmacovigilance; however, none of the reviews found rigorous evaluations of the reliability and validity of the data.

As this is a rapidly evolving field, we conducted a comprehensive scoping review to assess the utility of social media data for detecting adverse events related to health products, including pharmaceuticals, medical devices, and natural health products.

## Methods

### Research questions

The specific research questions were:

(1) Which social media listening platforms exist to detect adverse events related to health products, and what are their capabilities and characteristics?

(2) What is the validity and reliability of data from social media for detecting these adverse events?

### Study design

We used a scoping review method to map the concepts and types of evidence that exist on pharmacovigilance using social media data [8]. Our approach followed the rigorous scoping review methods manual by the Joanna Briggs Institute [9].

### Protocol

The Preferred Reporting Items for Systematic Reviews and Meta-analysis Protocols (PRISMA-P) [10] guideline was used to develop our protocol, which we registered with the Open Science Framework [11] and published in a peer-reviewed journal [12]. The protocol was developed by the research team and approved by members of the Health Canada Health Products and Food Branch, the commissioning agency of this review. Since the full methods have been published in the protocol [12], they are briefly outlined below.

### Eligibility criteria

The eligibility criteria were any type of document (e.g., journal article, editorial, book, webpage) that described listening to social media data for detecting adverse events associated with health products (see Additional file 1: Appendix 1). The following interventions were excluded from our review: programs of care, health services, organization of care, as well as public health programs and services. Documents related to the mining of social media data to detect prescription drug misuse and abuse were eligible for inclusion. *Social media listening* was defined as mining and monitoring of user-generated and crowd-intelligence data from online conversations in blogs, medical forums, and other social networking sites to identify trends and themes of the conversation on a topic (see Additional file 1: Appendix 2). We included documents that reported on at least one of the following outcomes: social media listening approaches, utility of social media data for pharmacovigilance and their performance capabilities, validity and reliability of user-generated data from social media for pharmacovigilance, and author’s perception of utility and challenges of using social media data.

### Information sources and search strategy

Comprehensive literature searches were conducted in MEDLINE, EMBASE, and the Cochrane Library by an experienced librarian. The MEDLINE search strategy was peer-reviewed by another librarian using the PRESS checklist [13], which has been published in our protocol [12], and also available in Additional file 1: Appendix 3. In addition, we searched grey literature (i.e., difficult to locate, unpublished documents) sources outlined in Additional file 1: Appendix 4 using the Canadian Agency for Drugs and Technologies in Health guide [14], and scanned the reference lists of relevant reviews [5, 6, 15].

### Study selection process

After the team achieved 75% agreement on a pilot-test of 50 random citations, each citation was independently screened by reviewer pairs (WZ, EL, RW, PK, RR, FY, BP) using Synthesi.SR; an online application developed by the Knowledge Translation Program [16]. Potentially relevant full-text documents were obtained and the same process (described above) was followed for full-text screening.

### Data items and data abstraction process

Data were abstracted on document characteristics (e.g., type of document), population characteristics of social media users (e.g., disease), characteristics of social media data (e.g., social media source), characteristics of social media listening approaches (e.g., pre-processing), and performance of the different approaches (e.g., validity and reliability of social media data). After the team pilot-tested the data abstraction form using a random sample of 5 included documents, each document was abstracted by one reviewer (WZ, EL, RW, PK, RR, FY, BP) and verified by a second reviewer (WZ, EL). The data were cleaned by a third reviewer (WZ, EL) and confirmed by the content expert (SJ, GH).

### Risk of bias assessment or quality appraisal

Risk of bias or quality appraisal was not conducted, which is consistent with the Joanna Briggs Institute methods manual [9], and those documented in scoping reviews on health-related topics [17].

### Synthesis of results

To characterize the health conditions studied, the World Health Organization version of the International Statistical Classification of Diseases and Related Health Problems (10th Revision, ICD-10) was used [18]. The social media system characteristics were described and categorized according to the steps typically involved in a social media data processing pipeline [19]. In addition, the social media systems were classified according to whether they were manual systems (i.e., coded by hand, without computer assistance), experimental/developmental stage systems (i.e., automatic information extraction systems being developed by researchers), or fully developed systems (i.e., automatic information extraction systems that are either commercially available or being used by regulatory agencies).

Descriptive statistics were performed (e.g., frequencies, measures of central tendency) using Excel 2010. Thematic analysis of open-text data was performed by two reviewers (WZ, EL) and verified by a third reviewer (ACT or SJ) to categorize the author perception of utility and challenges of using social media data for pharmacovigilance [20].

## Results

### Study flow

**Fig. 1:**
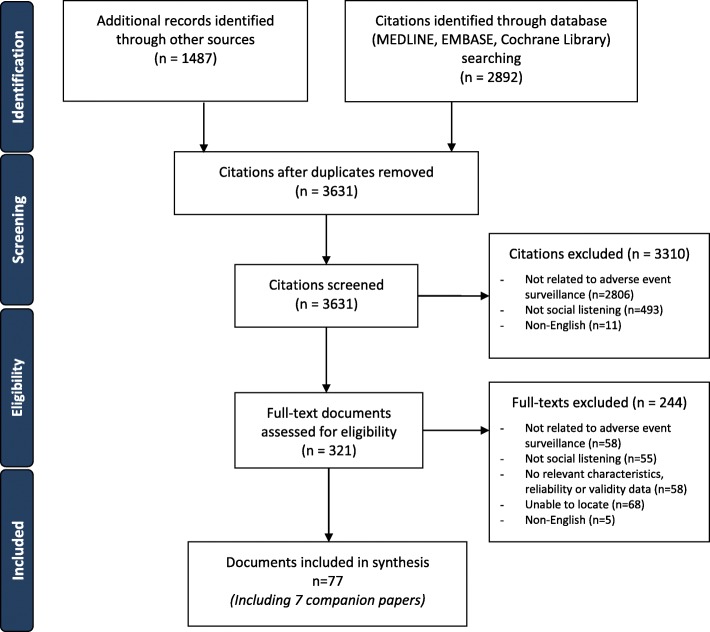
Study flow diagram

A total of 3631 citations from electronic databases and grey literature and other sources (e.g., reference scanning) were screened (Fig. 1). Of these, 321 potentially relevant full-text records were screened and 70 unique records with an additional 7 companion reports were included in our scoping review. The full list of included documents and companion reports can be found in Additional file 1: Appendix 5.

### Document characteristics

The documents were dated between 2001 and 2016 with 78% of the relevant documents being from 2013 onwards (Table 1). The most common document types were journal articles (57%) and conference papers (33%). Most of the corresponding authors were from North America (73%) and Europe (17%). Public sources of funding were the most common (40%). Most of the papers (66%) described an experimental/developmental automated information extraction system to mine data from social media for drug safety surveillance.

**Table 1:** Document characteristics

Document characteristics (*n* = 70)		Count (%)
Year of dissemination	2001–2004	1 (1.4%)
	2005–2008	1 (1.4%)
	2009–2012	13 (18.6%)
	2013–2016	55 (78.6%)
Document type	Blog	1 (1.4%)
	Dissertation	1 (1.4%)
	Book section	2 (2.9%)
	Report	3 (4.3%)
	Conference paper/poster	23 (32.9%)
	Journal article	40 (57.1%)
Geographic region of publication	Asia	2 (2.9%)
	Australia & New Zealand	5 (7.1%)
	Europe	12 (17.1%)
	North America	51 (72.9%)
Funding type	Non-sponsored	4 (5.7%)
	Industry and public-sponsored	5 (7.1%)
	Industry-sponsored	7 (10.0%)
	Not reported	26 (37.1%)
	Public-sponsored	28 (40.0%)
Types of social media listening systems studied for drug safety surveillance	Used an available automatic information extraction system (fully developed and available for use)	8 (11.4%)
	Used a manual approach for information extraction	16 (22.9%)
	Used an experimental automatic information extraction system (at the development stage)	46 (65.7%)

### Social media data characteristics

**Fig. 2:**
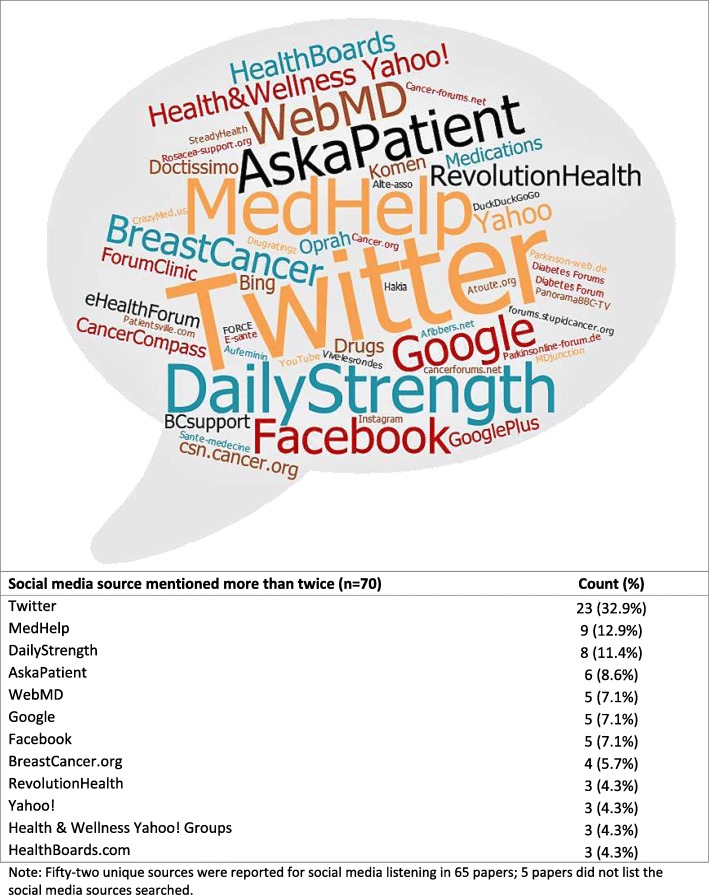
Wordcloud of social media sources mined in the documents

The commonly mined sources of social media platforms were Twitter (33%), MedHelp (13%), DailyStrength (11%), and AskaPatient (9%) (Fig. 2). The majority of the documents mined only one social media site to obtain user-generated data (54%) (Table 2). The user types included patients from health forums, such as BreastCancer.org (50%); the general population on micro-blogging sites, such as Twitter (39%); or both (10%). The geographic location of the social media users was seldom reported (17%). When it was reported, the users were from high-income countries as per the World Bank classification [21]. The social media posts were mostly in English (86%), followed by Spanish, French, and multiple languages (3% each). A small fraction (1% each) of posts was written in German and Serbian.

**Table 2:** Social media data characteristics

Social media data characteristics (*n* = 70)		Count (%)
Number of social media sources included by study authors	1	38 (54.3%)
	2	10 (14.3%)
	3	8 (11.4%)
	4	3 (4.3%)
	> 5	7 (10.0%)
	Not reported	4 (5.7%)
Type of social media sites	Patient-specific	35 (50.0%)
	General population	27 (38.6%)
	Both patient-specific and general population	7 (10.0%)
	Not reported	1 (1.4%)
Region of origin of the social media posts	USA	5 (7.1%)
	Spain	2 (2.9%)
	Germany	1 (1.4%)
	50+ countries	1 (1.4%)
	USA, Canada, UK, Australia, New Zealand	1 (1.4%)
	France	1 (1.4%)
	UK, North America, Australasia	1 (1.4%)
	Not reported	58 (82.9%)
Language of the social media posts	English	60 (85.7%)
	Spanish	2 (2.9%)
	French	2 (2.9%)
	German	1 (1.4%)
	Serbian	1 (1.4%)
	Multilingual	2 (2.9%)
	Not reported	2 (2.9%)
Method employed to collect social media data	Web crawling/ spidering software	19 (27.1%)
	API of the host site	14 (20.0%)
	Keyword search	7 (10.0%)
	Multiple methods	4 (5.7%)
	Other methods	13 (18.6%)
	Not Reported	13 (18.6%)
Duration (years) of social media data	Median (Q1, Q3)	1.13 (0.5, 7.13)
	Not reported	34 (48.6%)
Number of social media posts retrieved	Median (Q1, Q3)	42,594 (4608, 711,562)
	Not reported	5 (7.1%)

The posts were collected for a median duration of 1.13 years (i.e., the investigators “listened” to social media conversations for this duration), with an interquartile range of 6 months to 7 years. A median of 42,594 posts were included in the documents, with an interquartile range of 4608 to 711,562 posts. A variety of techniques were used to identify relevant social media posts, such as web crawlers or spiders (27%, i.e., an automated program that scans the social media source to identify posts about adverse events), application programming interfaces (APIs) of the host site (20%, i.e., a set of applications, rules, and definitions used to build the data set) and keyword search of social media sites (10%). Four papers (6%) used a combination of the above methods. Other approaches included using browser add-ons to monitor search query activities, using a pre-existing database of social media conversations, and requesting processed database from the social media site system administrators.

### Health conditions, types of surveillance, and types of health products

The majority of the documents applied their social media listening approach to the study of a specific health condition (Table 3). According to the ICD-10 classification system, almost half (46%) included patients with health conditions from more than one disease system, 13% included patients with neoplasms, and 11% included patients with mental illness and behavioural disorders. The focus of surveillance was most commonly any adverse event (74%), and the type of health products examined were mostly pharmaceutical drugs (98.6%).

**Table 3:** Health conditions, types of surveillance, and types of health products investigated

Topic (*n* = 70)		Count (%)
Health conditions studied as per ICD-10	Multiple disease system	32 (45.7%)
	Neoplasm	9 (12.9%)
	Mental illness & behavioural disorders	8 (11.4%)
	Factors influencing health status & contact with health services	6 (8.6%)
	Nervous system	6 (8.6%)
	Endocrine, nutritional & metabolic	3 (4.3%)
	Not reported	2 (2.9%)
	Circulatory system	2 (2.9%)
	Injury, poisoning and certain other consequences of external causes	1 (1.4%)
	Skin & subcutaneous tissue	1 (1.4%)
Type of surveillance studied	Any adverse event	52 (74.3%)
	Drug abuse/misuse	6 (8.6%)
	Drug-to-drug interaction	4 (5.7%)
	Specific adverse event (e.g., arthralgia, heart diseases, infertility)	7 (10.0%)
	Treatment switching	1 (1.4%)
Type of health products included	Pharmaceutical drugs (including biologics)	69 (98.6%)
	Medical devices	1 (1.4%)
	Natural health products	0 (0.0%)

### Social media data processing pipeline

A variety of data processing approaches were identified (Additional file 1: Appendix 6):

- supervised learning (21%, i.e., a machine learning approach that is trained from a set of labeled data that has been coded typically by humans)
- rule-based learning (9%, i.e., a learning algorithm that allows automatic identification of useful rules, rather than a human needing to apply prior domain knowledge to manually constructed rules and curate a rule set)
- semi-supervised learning (7%, i.e., a machine learning method that uses both labeled and unlabeled data for training)
- unsupervised learning (6%, i.e., machine learns patterns in the data without any labels given by humans)

**Fig. 3:**
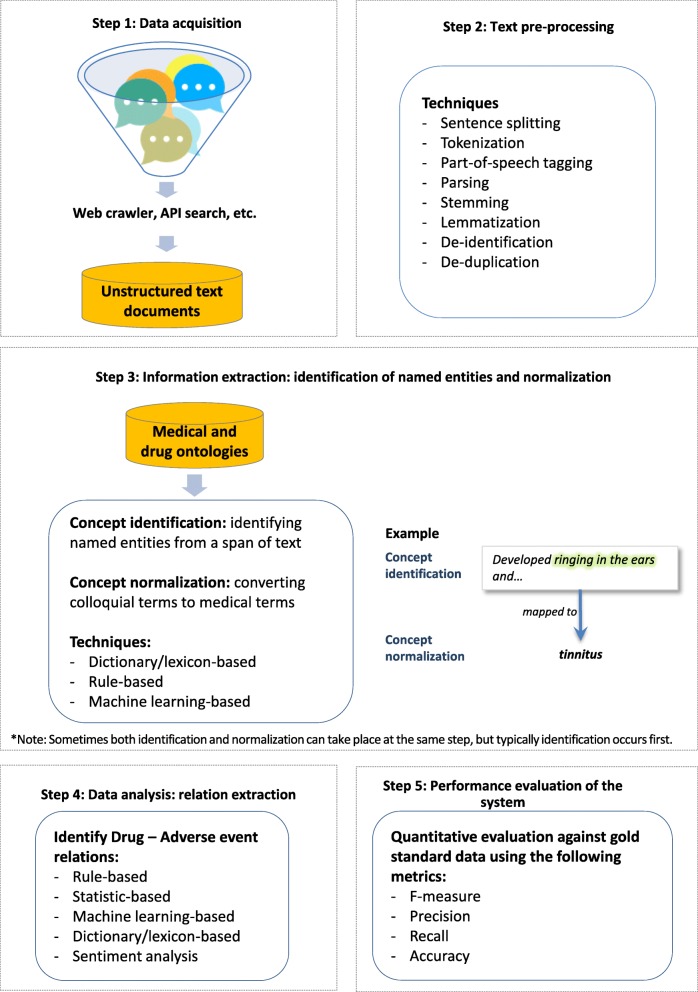
Steps typically involved in social media data processing flow

After the social media dataset was created, the following steps were typically used to process the social media data (Fig. 3):

1. pre-processing (e.g., removing punctuations and stop words; breaking text into words, phrases, and symbols or tokens; reducing words to the root; and correcting spelling mistakes)
2. de-identification (e.g., removing identifiable information, such as user names and addresses)
3. de-duplication (e.g., removing duplicate and related posts)
4. concept identification (e.g., identifying adverse drug reactions and other events from a sequence text)
5. concept normalization (e.g., converting colloquial terms to medical terms for drug names, symptoms, history, events, disease)
6. relation extraction (e.g., determining the relationship between the health product and an adverse event)

Different text processing methods were reported for each step, with varying levels of automation.

### Types of social media listening systems

Most of the documents examined an experimental social media listening system that was under development (66%). Seven pre-existing social media listening systems were identified in eight documents (Additional file 1: Appendix 7): MedWatcher Social (US Food and Drug Administration), commercial systems AETracker (IMS health), Visible Intelligence (Cision), BeFree System: Bio-Entity Finder & RElation Extraction (Integrative Bioinformatics group), MeaningCloud (MeaningCloud LLC, Sngular company), Treato (Treato Ltd.), and OpenCalais (Thomson Reuters). An eighth system was identified during the environmental scan, which is known as the Web-RADR Social and is currently under development by European Union regulators. Sixteen documents studied a manual approach, whereby posts were coded manually by humans without any assistance from computers (23%).

### Outcome results

#### Utility of social media data for pharmacovigilance

Nineteen documents provided information on the utility of social media data for pharmacovigilance; however, this was not considered to be the primary objective of the documents (Additional file 1: Appendix 8). Most of the documents focused on the detection of adverse events, while one document focused on the timing of adverse event detection [22].

Ten documents compared social media data against spontaneous reports to regulatory agencies to study the difference in the number of adverse events captured, time lag in detecting adverse events or whether the two data sources are correlated [22–31]. Specifically, 7/10 documents [25–31] compared the frequency of adverse events detected from social media source(s) versus a regulatory database, and all but one [26] reported consistent results [24–30]. In 2/10 documents, authors reported a positive correlation between adverse events reported in social media data sources and those reported by regulatory agencies [23, 31]. In one document, timing of adverse event reporting on social media was compared with the timing of the FDA’s official alert or labeling revision time, and adverse events were detected on social media earlier [22].

Six documents compared adverse events reported in social media posts against published data or safety signals known by the authors, [32–38] (exact sources were not specified) and all found consistent results. One document compared weighted scores of adverse events reported in social media posts with drugs withdrawn from the market and found a positive correlation between higher weighted scores and withdrawn drugs [39]. One document compared the frequency of adverse events reported on social media with those from a large integrated healthcare system database and found that their results were generally consistent, though several less frequently reported adverse events in the medical health records were more commonly reported on social media (e.g., aspirin-induced hypoglycemia was discussed only on social media) [40]. In contrast, one document found that less than 2% of adverse events detected by AETracker (a commercially available platform) were actual events, as confirmed by pharmacovigilance experts [41].

#### Themes of utility and challenges of social media listening

Several themes emerged from the qualitative analysis of the study authors' discussions of the strengths and limitations of using social media data for pharmacovigilance. In order of prevalence, the utility of social media listening were as follows: provides supplemental data to traditional surveillance systems, captures perceptions about the effects of treatment (including adverse events), and offers an extensive source of publicly available data (Table 4). The three most common challenges were the unstructured nature of the data, complex structure of the text data, and potential lack of representativeness (i.e., posts may not be representative of all those administered health products) (Table 4). Further details can be found in Additional file 1: Appendix 17.

**Table 4:** Utility and challenges of social media listening

Utility and challenges of social media listening	Count (%)
Utility of social media listening for pharmacovigilance	
Supplemental data to traditional post-marketing safety surveillance	31 (44.3%)
Captures perceptions and consequences of treatment and adverse events	14 (20.0%)
Large publicly available data source	14 (20.0%)
Able to discover undocumented or rare adverse events	11 (15.7%)
Promising early warning system	10 (14.3%)
Computationally efficient	7 (10.0%)
Captures prescription drug misuse/abuse	4 (5.7%)
Not biased towards severe adverse events	7 (10.0%)
Captures large geographical area	3 (4.3%)
Useful for risk communication	3 (4.3%)
Able to extract complex medical concepts	2 (2.9%)
Can be more accurate than spontaneous reporting systems	2 (2.9%)
Hypothesis-generating	2 (2.9%)
Able to identify undocumented drug interactions	2 (2.9%)
Findings are similar to traditional systems	1 (1.4%)
Captures information on adherence related to adverse events	1 (1.4%)
Challenges of social media listening for pharmacovigilance	
Non-standard reporting format (informal language, format used to report information, amount of information provided by each user)	30 (42.9%)
Difficult to draw complex semantic relationships from unstructured texts	14 (20.0%)
May not be a representative population	13 (18.6%)
Noise may exist in signal detection	12 (17.1%)
Inadequate information to draw causality	9 (12.9%)
Lacks comprehensive medical and demographic information	8 (11.4%)
Subjective, incomplete or misinformation	6 (8.6%)
Not a balanced coverage of all drugs and medical conditions	5 (7.1%)
Data acquisition challenges due to host site restrictions	4 (5.7%)
Duplication of data (double-counting)	4 (5.7%)
Processing multi-lingual texts	3 (4.3%)
Resource-intensive to process big data	2 (2.9%)

#### Validity and reliability of analytics used to process social media data

The validity and reliability measures were categorized according to the social media data processing pipeline, as follows:

##### Pre-processing

Thirty-two documents reported methods for pre-processing (Additional file 1: Appendix 9), and provided information on the software used and accessibility of the tool (e.g., public, proprietary) [23, 26, 32, 34, 35, 42–72]. Validity or reliability of data processed in this step was not reported.

##### De-identification

Six documents reported methods for de-identification (Additional file 1: Appendix 10), and provided information on the software used and accessibility of the tool [31, 43, 53–56, 64, 73]. Only one study reported validity/reliability of data processed in this step, which included a precision of 67%, recall of 98%, and F-measure of 80% [43].

##### De-duplication

Five documents reported methods for de-duplication (Additional file 1: Appendix 11), and provided information on the software used and accessibility of the tool [30, 40, 74–76]. Validity or reliability of data processed in this step was not reported.

##### Concept identification

Forty-five documents reported on automated methods for concept identification for drugs, adverse events, and overall (i.e., drug and adverse events), which were reported using the following approaches: dictionary/lexicon-based (*n* = 30) [22, 23, 26, 30, 31, 39, 40, 43, 44, 47, 50, 51, 53–57, 59, 63–69, 72, 74, 75, 77–82], supervised classifier (*n* = 6) [42, 61, 62, 71, 73, 83], mixed lexicon-based/supervised classifier (*n* = 2) [52, 58], rule-based phrase extraction (*n* = 2) [34, 35, 84], sentiment analysis (*n* = 2) [32, 46], statistical approaches (*n* = 2) [58, 70], or unspecified (*n* = 1; Additional file 1: Appendix 12) [85]. Three documents reported evaluation results for overall concept identification. In these, supervised classifier approaches were studied and their accuracy for overall concept identification ranged from 78 to 83%, precision ranged from 32 to 78%, recall ranged from 32 to 74%, and F-measure ranged from 42 to 61%. [62, 71, 73] Validity and reliability results for drug and adverse event concepts can be found in Additional file 1: Appendix 14.

##### Concept normalization – Drug names

Nineteen documents reported on automated methods for concept normalization of drug names and all used a variation of dictionary or lexicon-based approaches (*n* = 19; Additional file 1: Appendix 13) [22, 30, 43, 47–51, 54–58, 63–66, 74, 75, 78, 79, 83, 86]. One paper also used a hybrid approach of statistical modeling using conditional random fields and dictionary-based methods [58]. Two documents reported accuracy results for dictionary/lexicon-based approaches, which ranged from 0 to 92% [58, 83]. Using a hybrid approach of statistical methods combined with dictionary-based methods, Metke-Jimenez and colleagues found accuracy results ranging from 75 to 77% [58].

##### Concept normalization – Medical events

Thirty-four documents reported on automated methods for concept normalization of medical concepts (i.e., adverse events, symptoms, disease), of which 33 documents reported a dictionary/lexicon-based approach [22, 23, 26, 30–32, 39, 40, 43–45, 48, 50, 51, 53–59, 61, 63, 64, 66–69, 71, 72, 74, 75, 77, 81–83], and 2 documents also used statistical approaches (Additional file 1: Appendix 14) [58, 70]. Two documents reported accuracy of dictionary/lexicon approaches, which ranged from 3 to 67%. Using a hybrid approach of statistical methods combined with dictionary methods, Metke-Jimenez and colleagues found accuracy results ranging from 33 to 38%.

##### Relation extraction

Thirty-eight documents reported on automated methods for relation extraction, which is used to establish relationships between drugs and adverse events using social media data (Additional file 1: Appendix 15). Methods were classified as rule-based or statistical association mining (*n* = 16) [22, 31, 34, 35, 40, 43, 47, 51, 60, 67–69, 75–77, 83, 85], supervised classifier (*n* = 16) [30, 39, 44, 49, 53–57, 61, 63–66, 70, 71, 73, 74, 86], dictionary/lexicon based (*n* = 4) [23, 45, 78–80] or sentiment analysis (*n* = 2) [32, 46]. Eight documents provided validity/reliability data on rule-based or statistical association mining. The precision ranged from 35 to 79%, recall ranged from 6 to 100%, F-measure ranged from 9 to 94%, and area under the curve (AUC) ranged from 0.57 to 0.93. Fifteen documents provided validity and reliability data for the supervised classifier approach and accuracy ranged from 29 to 90%, precision from 20 to 86%, recall from 23 to 100%, F-measure from 32 to 92%, and AUC was 74%. Two documents provided data on the reliability and validity of dictionary/lexicon-based approaches, which ranged from 44 to 83% for precision, 2 to 84% for recall, and 3 to 58% for the F-measure. Validity and reliability of data for this step were not reported for sentiment analysis or semantic matching approaches.

##### Additional processing steps

Some authors investigated additional processing steps, which included identifying the source of an adverse event report (*n* = 4; i.e,. personal experience vs. witnessed) [42, 44, 53–56], and query matching which retrieves and filters the relevant documents to answer a given user query (*n* = 1; Additional file 1: Appendix 16) [87]. Eight documents analysed user-generated texts in other languages, including Spanish [78–80], French [29, 88], German [37], Serbian [51], and multiple languages [45, 77] but only 5 reported their methods [47, 52, 62, 88–90]. One document included 53 different languages [77] and another document included English, Spanish, and French [45]. None of the papers described validity or reliability of processing non-English text.

## Discussion

Most of the documents included in our scoping review dated from 2013 onwards. We identified seven pre-existing social media platforms, and another platform (Web-RADR Social) that is currently under development by European regulators. Unfortunately, no information on when this social media platform will be completed was provided. The majority of the documents primarily focused on the development of social media listening tools for pharmacovigilance (as opposed to their application), which would be useful for those interested in developing such platforms. In particular, documents authored by Freifeld et al., [74] Karimi et al., [83] and Vinod et al. [19] provide useful information on the development of such platforms.

We identified 19 documents providing some information on the utility of social media. This information was mostly abstracted from the discussion section of the documents, suggesting that the conclusions were highly speculative. Furthermore, most of the included documents only followed social media posts for a median duration of 1 year. A high-quality study that examines utility over a longer timeframe with a broader data frame may provide further useful information to the field.

According to authors’ perceptions, social media can be used to supplement traditional reporting systems, to uncover adverse events less frequently reported in traditional reporting systems, to communicate risk and to generate hypotheses. However, challenges exist, such as difficulties interpreting relationships between the drugs and adverse events (e.g., there are inadequate data to draw causality), potential lack of representativeness between social media users and the general population, and the resource-intensive process of using social media data for pharmacovigilance. Evaluation studies of pharmacovigilance using social media listening are needed to substantiate these perceptions. Future studies should also consider evaluating the performance and utility of integrating social media data with other data sources, such as regulatory databases that collect spontaneous reports, as well as relevant surveillance databases.

Our results have summarized the most common elements involved in the processing of social media data for pharmacovigilance. Across the included documents, the most common steps employed were: 1) pre-processing; 2) de-identification; 3) de-duplication; 4) concept identification; 5) concept normalization; and 6) relation extraction. Validity and reliability findings varied across the different approaches that were used to mine the data, which suggests some may be more effective than others. The heterogeneous nature of the study designs and approaches reported in the documents; however, make it difficult to definitively determine which approaches are more useful than others.

As described in our protocol, we conducted this scoping review to inform members of Health Canada who are currently using our results to plan an evaluation study on utility of social media for detecting health product adverse events. They may also consider a Canadian platform to be developed in the future, depending on the results of their study.

Our results are similar to 3 other reviews on this topic. A recent review by Sarker et al. [7] described the different automatic approaches used to detect and extract adverse drug reactions from social media data for pharmacovigilance purposes in studies published in the last 10 years. Although the authors characterized existing social media listening and analytics platforms, validity and reliability of the user-generated data captured through social media and crowd-sourcing networks were not examined. Golder and colleagues [5] conducted a systematic review on adverse events data in social media. They found that although reports of adverse drug events can be identified through social media, the reliability or validity of this information has not been formally evaluated. Finally, Lardon and colleagues [6] conducted a scoping review on the use of social media for pharmacovigilance. They identified numerous ways to identify adverse drug reaction data, extract data, and verify the quality of information. However, gaps in the field were identified. For example, most studies identifying adverse drug reactions failed to verify the reliability and validity of the data and none of the studies proposed a feasible way to integrate data from social media across more than one site/information source.

The strengths of our scoping review include a comprehensive search of multiple electronic databases and sources for difficult to locate and unpublished studies (or grey literature), as well as the use of the rigorous scoping review methods manual by the Joanna Briggs Institute. In addition, we included researchers with computer science expertise (SJ, GH) to help code automated approaches. In terms of a dissemination plan, we will use a number of strategies, such as: a 1-page policy brief, two stakeholder meetings (i.e., consultation exercises), presentations at an international conference, and publications in open-access journals. Team members will also use their networks to encourage broad dissemination of results.

There are some limitations to our scoping review process. The review was limited to documents written in English to increase its feasibility, given the 5-month timeline. Additionally, due to the large number of documents identified, the data were abstracted by one reviewer and verified by a second reviewer. Lastly, although our literature search was comprehensive, there is always a chance that some social media platforms or data analytics documents were missed. Since this is a rapidly evolving and emerging field, we expect that new documents fulfilling our inclusion criteria will be released in increasing numbers [91, 92], highlighting a potential need to update our review in the near future.

## Conclusion

Our results suggest that the use of social media is being investigated for drug safety surveillance from an early developmental perspective. Within this context, social media data has the potential to supplement data from regulatory agency databases, capture less frequently reported AEs, and identify AEs earlier than official alerts or regulatory changes. However, the utility, validity and implementation of information extraction systems using social media for pharmacovigilance are under-studied. Further research is required to strengthen and standardize the approaches as well as to ensure that the findings are valid, for the purpose of pharmacovigilance.

## Additional file

Additional file 1: Appendix 1. Description of included and excluded interventions for drug safety surveillance. Appendix 2. Glossary of terms. Appendix 3. Medline search strategy. Appendix 4. Sources for grey literature search. Appendix 5. List of included studies. Appendix 6. Social media data processing pipeline. Appendix 7. Pre-existing social media listening platforms for drug safety surveillance. Appendix 8. Utility of social media data for drug safety surveillance. Appendix 9. Pre-processing methods and results. Appendix 10. De-identification methods and results. Appendix 11. De-duplication methods and results. Appendix 12. Concept identification methods and results. Appendix 13. Drug name normalization methods and results. Appendix 14. Medical event normalization methods and results. Appendix 15. Relation extraction methods and results. Appendix 16. Additional processing methods and results. Appendix 17. Utility and challenges with socila media listening for drug safety surveillance. (DOCX 275 kb)

## Abbreviations

AE: adverse events
API: application programming interfaces
AUC: area under the curve
FDA: food and drug administration
ICD: International Statistical Classification of Diseases and Related Health Problems
PRISMA-P: Preferred Reporting Items for Systematic Reviews and Meta-analysis Protocol
