# Supplementary material for: Utility of social media and crowd-intelligence data for pharmacovigilance: a scoping review
Source: BMC Med Inform Decis Mak. 2018 Jun 14;18:38. doi: 10.1186/s12911-018-0621-y (PMC6001022; doi:10.1186/s12911-018-0621-y)
Supplement: Supplementary file 1 — Appendix 1. Description of included and excluded interventions for drug safety surveillance. Appendix 2. Glossary of terms. Appendix 3. Medline search strategy. Appendix 4. Sources for grey literature search. Appendix 5. List of included studies. Appendix 6. Social media data processing pipeline. Appendix 7. Pre-existing social media listening platforms for drug safety surveillance. Appendix 8. Utility of social media data for drug safety surveillance. Appendix 9. Pre-processing methods and results. Appendix 10. De-identification methods and results. Appendix 11. De-duplication methods and results. Appendix 12. Concept identification methods and results. Appendix 13. Drug name normalization methods and results. Appendix 14. Medical event normalization methods and results. Appendix 15. Relation extraction methods and results. Appendix 16. Additional processing methods and results. Appendix 17. Utility and challenges with socila media listening for drug safety surveillance. (DOCX 275 kb) [file 12911_2018_621_MOESM1_ESM.docx]

**Additional File 1. Appendices**

[Appendix 1. Description of included and excluded interventions for health product safety surveillance 2](#_Toc496186580)

[Appendix 2. Glossary of Terms 3](#_Toc496186581)

[Appendix 3. Medline search strategy 5](#_Toc496186582)

[Appendix 4. Sources for grey literature search 6](#_Toc496186583)

[Appendix 5. List of included studies 15](#_Toc496186584)

[Appendix 6. Social media data processing pipeline 21](#_Toc496186585)

[Appendix 7: Pre-existing social media listening platforms for drug safety surveillance 23](#_Toc496186586)

[Appendix 8. Utility of social media data for pharmacovigilance 24](#_Toc496186587)

[Appendix 9. Pre-processing methods and results 26](#_Toc496186588)

[Appendix 10. De-identification methods and results 29](#_Toc496186589)

[Appendix 11. De-duplication methods and results 30](#_Toc496186590)

[Appendix 12. Concept identification methods and results 31](#_Toc496186591)

[Appendix 13. Drug name normalization methods and results 43](#_Toc496186592)

[Appendix 14. Medical event normalization methods and results 47](#_Toc496186593)

[Appendix 15. Relation extraction methods and results 53](#_Toc496186594)

[Appendix 16. Additional processing methods and results 62](#_Toc496186595)

[Appendix 17. Utility and challenges of social media data for pharmacovigilance 64](#_Toc496186596)

[References 70](#_Toc496186597)

# Appendix 1. Description of included and excluded interventions for health product safety surveillance

| Included interventions |  |
| --- | --- |
| Pharmaceuticals and drug products (including biologics) | Include prescription and non-prescription (over-the-counter) medicines, disinfectants and sanitizers with disinfectant claims and biologics such as, vaccine, serums, blood-derived products, hormones, growth factors and enzymes manufactured in bacterial, yeast or mammalian cell lines; and gene therapy and cell therapy products.[1, 2] |
| Medical devices | Include defibrillators, syringes, surgical lasers, hip implants, medical laboratory diagnostic instruments (including X-ray, ultrasound devices), contact lenses, and condoms.[3] |
| Natural health products | Include vitamins and minerals, herbal remedies, homeopathic and traditional medicines, probiotics, and other products like amino acids and essential fatty acids.[4] |
| Excluded interventions |  |
| Program of care | Any evidence-based health care delivery plans that describe treatment shown to be effective for specific injuries and illnesses, such as musculoskeletal injury, mild traumatic brain injuries.[5] |
| Health services | Include all services dealing with the diagnosis and treatment of disease, or the promotion, maintenance and restoration of health. They include personal and non-personal health services.[6] |
| Organizations of care | Any establishment offering health care service such as hospitals, walk-in clinics. |
| Public health programs and services | Any programs or services targeted for population health improvement such as health education programs (e.g. sexual health clinics), health promotion strategies (e.g. physical activity), disease surveillance and health screening (e.g. breast cancer screening, disease outbreaks). |

# Appendix 2. Glossary of Terms

| Glossary of terms | |
| --- | --- |
| Accuracy | A measure for information retrieval to determine the fraction of classifications that are correct |
| Application program interface (API) | A set of routines, protocols and definitions that describe and prescribe the expected behavior or specification of a program |
| Area under the curve (AUC) | A measure for model comparison that quantifies the probability that a classifier will rank a randomly chosen positive instance higher than a randomly chosen negative one (assuming 'positive' ranks higher) |
| Association mining | Uses statistical association and co-occurrence to infer rules that describe relationships in the data. Also known as association rule mining. |
| Conditional random field (CRF) | A sequence modeling framework that predicts a sequence of labels based on an observation sequence using conditional distributions learned from training data |
| Decision tree | A tree-based classification model that predicts the value of a target variable based on several input variables |
| Dependency parses | Analyzes the grammatical structure of a sentence, establishing relationships between words |
| Dictionary-based | A machine translation method that uses word matching based on pre-compiled dictionary entries |
| F-measure | The weighted harmonic mean of precision and recall used to measure a test’s accuracy |
| Fisher-Robinson model | Classifier that uses the inverse chi-square function to calculate class probabilities based on the frequency of the features in the data |
| Fuzzy matching | A technique used in machine translation that allows some character deviations when matching to a list |
| Graphical model | Information extracted by creating a graph that represents how different adverse events and drugs are related based on mentions in the text |
| Hidden markov model (HMM) | A model that uses a sequence of observations to determine the most probable underlying state that produced that sequence |
| Informedness | The probability that a classifier is making an informed decision versus pure guesswork |
| Lexicon-based | A method that searches for terms contained in a dictionary, which may include spelling variations |
| Maximum Entropy model (ME) | Multinomial logistic regression classifier that does not assume independence between features |
| Naive Bayes classifier (NB) | Classifier that learns to label input based on the “naive” assumption that all features are independent |
| Network analysis | Based on analyses of the links between drugs and symptoms using an automatically generated network |
| Phonetic matching | An algorithm for indexing words by their pronunciation |
| Precision | A measure for information retrieval to determine the fraction of retrieved instances that are relevant (also known as positive predictive value) |
| Random Forest model | A classifier based on a composite of multiple decision trees |
| Recall | A measure for information retrieval to determine the fraction of relevant instances that have been retrieved (also known as sensitivity) |
| Rocchio | Assigns new data points to the class with the closest center point |
| Semantic clustering | A technique to group source artifacts that use similar vocabulary |
| Sentiment analysis | A method to detect favorable and unfavorable opinions from text documents |
| Sliding window | A method of text matching that considers all possible phrases of a fixed length (the window size) |
| Spider | An automated program that scans social media sites and creates entries with searchable indexes (also known as web crawler) |
| Supervised classifier | Machine learning classifier trained on labeled data and tested on separate data |
| Support vector machine (SVM) | Classifier that maps input to a higher-dimensional vector space in order to find a decision boundary between two classes of data |
| Latent Dirichlet allocation | A topic modeling algorithm that generates topics based on word frequency from a set of documents |

# Appendix 3. Medline search strategy

**Interface:** Ovid MEDLINE(R) Epub Ahead of Print, In-Process & Other Non-Indexed Citations, Ovid MEDLINE(R) Daily and Ovid MEDLINE(R) 1946 to Present

| 1. social media/ | | | | | | | | | | | | | | | | | | | | | | | | | | | | | | | | | | | |  | | |  |  |  |  |  |  |  |  |
| --- | --- | --- | --- | --- | --- | --- | --- | --- | --- | --- | --- | --- | --- | --- | --- | --- | --- | --- | --- | --- | --- | --- | --- | --- | --- | --- | --- | --- | --- | --- | --- | --- | --- | --- | --- | --- | --- | --- | --- | --- | --- | --- | --- | --- | --- | --- |
| 2. Social Networking/ | | | | | | | | | | | | | | | | | | | | | | | | | | | | | | | | | | | | | | | |  | | | |  |  |  |
| 3. blogging/ | | | | | | | | | | | | | | | | | | | | | | | | | | | | | | | | | |  |  |  |  |  |  |  |  |  |  |  |  |  |
| 4. Crowdsourcing/ | | | | | | | | | | | | | | | | | | | | | | | | | | | | | | | |  |  |  |  |  |  |  |  |  |  |  |  |  |  |  |
| 5. (social adj3 (media or medium* or network* or bookmark*)).tw. | | | | | | | | | | | | | | | | | | | | | | | | | | | | | | | | | | | | | | | | |  |  |  |  |  |  |
| 6. (blog* or microblog*).tw. | | | | | | | | | | | | | | | | | | | | | | | | | | | | | |  |  |  |  |  |  |  |  |  |  |  |  |  |  |  |  |  |
| 7. ((patient or discussion or web or chat or internet or online) adj3 (forum* or fora or message board*)).tw. | | | | | | | | | | | | | | | | | | | | | | | | | | | | | | | | | | | | | | | | | | | | |  | |
| 8. (facebook or twitter or wiki or youtube or web 2* or instagram or foursquare or linkedin or pinterest or lifestream*).tw. | | | | | | | | | | | | | | | | | | | | | | | | | | | | | | | | | | | | | | | | | | | | |  | |
| 9. (crowdsourc* or crowd sourc*).tw. | | | | | | | | | | | | | | | | | | | | | | | | | | | |  |  |  |  |  |  |  |  |  |  |  |  |  |  |  |  |  |  |  |
| 10. or/1-9 | | | | | | | | | | | | | | | | | | | | | | | | | |  |  |  |  |  |  |  |  |  |  |  |  |  |  |  |  |  |  |  |  |  |
| 11. product surveillance, postmarketing/ or adverse drug reaction reporting systems/ or pharmacovigilance/ | | | | | | | | | | | | | | | | | | | | | | | | | | | | | | | | | | | | | | | | | | | | |  | |
| 12. exp "Drug-Related Side Effects and Adverse Reactions"/ | | | | | | | | | | | | | | | | | | | | | | | |  |  |  |  |  |  |  |  |  |  |  |  |  |  |  |  |  |  |  |  |  |  |  |
| 13. (side effect* or (adverse adj3 (effect* or event* or reaction*))).tw. | | | | | | | | | | | | | | | | | | | | | |  |  |  |  |  |  |  |  |  |  |  |  |  |  |  |  |  |  |  |  |  |  |  |  |  |
| 14. (pharmacovigilance or ((postmarketing or post-marketing) adj3 surveillance)).tw. | | | | | | | | | | | | | | | | | | | | | | | | | | | | | | | | | | | | |  |  |  |  |  |  |  |  |  |  |
| 15. Patient Safety/ | | | | | | | | | | | | | | | | | | | |  |  |  |  |  |  |  |  |  |  |  |  |  |  |  |  |  |  |  |  |  |  |  |  |  |  |  |
| 16. ae.fs. | | | | | | | | | | | | | | | | | |  |  |  |  |  |  |  |  |  |  |  |  |  |  |  |  |  |  |  |  |  |  |  |  |  |  |  |  |  |
| 17. Pharmacoepidemiology/ | | | | | | | | | | | | | | | |  |  |  |  |  |  |  |  |  |  |  |  |  |  |  |  |  |  |  |  |  |  |  |  |  |  |  |  |  |  |  |
| 18. Medication Errors/ | | | | | | | | | | | | | |  |  |  |  |  |  |  |  |  |  |  |  |  |  |  |  |  |  |  |  |  |  |  |  |  |  |  |  |  |  |  |  |  |
| 19. Abnormalities, Drug-Induced/ | | | | | | | | | | | |  |  |  |  |  |  |  |  |  |  |  |  |  |  |  |  |  |  |  |  |  |  |  |  |  |  |  |  |  |  |  |  |  |  |  |
| 20. ci.fs. | | | | | | | | | |  |  |  |  |  |  |  |  |  |  |  |  |  |  |  |  |  |  |  |  |  |  |  |  |  |  |  |  |  |  |  |  |  |  |  |  |  |
| 21. (drug* or medication* or pharmaceutical* or medicine* or biologics or vaccine* or herb* or vitamin*).tw. | | | | | | | | | | | | | | | | | | | | | | | | | | | | | | | | | | | | | | | | | | | | |  | |
| 22. ((medical or health) adj2 (device* or equipment* or instrument* or supply or supplies)).tw. | | | | | | | | | | | | | | | | | | | | | | | | | | | | | | | | | | | | | | | | | | |  | | |  |
| 23. 21 or 22 | | | | | | | |  |  |  |  |  |  |  |  |  |  |  |  |  |  |  |  |  |  |  |  |  |  |  |  |  |  |  |  |  |  |  |  |  |  |  |  |  |  |  |
| 24. (safe* or harm* or toxicity).tw. | | | | | |  |  |  |  |  |  |  |  |  |  |  |  |  |  |  |  |  |  |  |  |  |  |  |  |  |  |  |  |  |  |  |  |  |  |  |  |  |  |  |  |  |
| 25. 23 and 24 | | | |  |  |  |  |  |  |  |  |  |  |  |  |  |  |  |  |  |  |  |  |  |  |  |  |  |  |  |  |  |  |  |  |  |  |  |  |  |  |  |  |  |  |  |
| 26. or/11-20,25 | |  |  |  |  |  |  |  |  |  |  |  |  |  |  |  |  |  |  |  |  |  |  |  |  |  |  |  |  |  |  |  |  |  |  |  |  |  |  |  |  |  |  |  |  |  |
| 27. 10 and 26 |  |  |  |  |  |  |  |  |  |  |  |  |  |  |  |  |  |  |  |  |  |  |  |  |  |  |  |  |  |  |  |  |  |  |  |  |  |  |  |  |  |  |  |  |  |  |

# **Appendix 4. Sources for grey literature search**

| **Spontaneous reporting systems** | |
| --- | --- |
| Australia | https://www.ebs.tga.gov.au/ebs/ADRS/ADRSRepo.nsf?OpenDatabase |
| Canada | http://www.hc-sc.gc.ca/dhp-mps/medeff/index-eng.php  http://www.hc-sc.gc.ca/dhp-mps/medeff/report-declaration/index-eng.php |
| European database of AE | http://www.adrreports.eu/en/ |
| EudraVigilance | http://eudravigilance.ema.europa.eu/ |
| UK Yellow Care | https://yellowcard.mhra.gov.uk  http://webarchive.nationalarchives.gov.uk/20141205150130/  http:/www.mhra.gov.uk/home/groups/pl-p/documents/websiteresources/con408250.pdf |
| US MedWatch | http://www.fda.gov/Safety/MedWatch/ |
| US Sentinel Initiative | http://www.fda.gov/Safety/FDAsSentinelInitiative/ucm2007250.htm |
| US FAERS | https://open.fda.gov/data/faers/ |
| Vigibase | http://www.who-umc.org/DynPage.aspx?id=98082&mn1=7347&mn2=7252&mn3=7322&mn4=7326 |
| **Associations** | |
| AIIM | http://www.aiim.org |
| American Health Information Management Association | http://www.ahima.org |
| CHIMA | https://www.echima.ca |
| CIHI | www.cihi.ca |
| COACH | http://www.coachorg.com/en/index.asp |
| The Institute of Electrical and Electronics Engineers, Incorporated | https://www.ieee.org/index.html |
| HIME | https://www.efmi.org/index.php/workinggroups/hime-health-information-management-europe |
| HIMSS | http://www.himss.eu |
| IMS Institute | http://www.imshealth.com/en/thought-leadership/ims-institute |
| IFHIMA | https://ifhima.org |
| IMIA | http://www.imia-medinfo.org/new2/ |
| Medicine for Europe | http://www.medicinesforeurope.com |
| UK Chip | http://www.ukchip.org |
| Health Informatics Associations and Societies | http://www.healthinformaticsforum.com/health-informatics-associations-and-societies |
| **Regulatory authority websites: Asia and Pacific** | |
| Australian Government: Department of Health and Ageing | http://www.health.gov.au/ |
| Australian Government: Therapeutic Goods Administration | http://www.tga.gov.au/ |
| Brunei: Ministry of Health | http://www.moh.gov.bn/Theme/Home.aspx |
| Fiji: Ministry of Health | http://www.health.gov.fj/ |
| Hong Kong: Department of Health | http://www.info.gov.hk/dh/ |
| India: Ministry of Consumer Affairs, Food & Public Distribution | http://mohfw.nic.in/ |
| India: Central Drug Standards Control Organisation | http://cdsco.nic.in/ |
| India: Ministry of Food and Consumer Affairs | http://fcamin.nic.in/ |
| Japan: Ministry of Health, Labour and Welfare | http://www.mhlw.go.jp/english/index.html |
| Japan: Pharmaceuticals and Medical Devices Evaluation Agency | http://www.pmda.go.jp/english/index.html |
| New Zealand: Ministry of Health | http://www.health.govt.nz/ |
| New Zealand: Medicines and Medical Devices Safety Authority | http://www.medsafe.govt.nz/ |
| New Zealand: Food Safety Authority | http://www.nzfsa.govt.nz/ |
| Papua New Guinea: Department of Health | http://www.health.gov.pg/ |
| Philippines: Department of Health | http://www.doh.gov.ph/ |
| Philippines: National Food Authority | http://www.nfa.gov.ph/ |
| Singapore: Ministry of Health | http://www.moh.gov.sg/mohcorp/default.aspx |
| Singapore: Health Sciences Authority | http://www.hsa.gov.sg/ |
| Singapore: Ministry of Environment  (food control) | http://www.env.gov.sg/ |
| Sri Lanka: Ministry of Health | http://www.health.gov.lk/ |
| Taiwan: Department of Health | http://www.doh.gov.tw/EN2006/index_EN.aspx |
| Thailand: Food and Drug Administration | http://www.fda.moph.go.th/eng/index.stm |
| Thailand: Ministry of Agriculture and Co-operatives | http://eng.moac.go.th/main.php?filename=index |
| **Regulatory authority websites: Europe** | |
| Belgium: Health, Food Chain Safety and Environment | http://www.health.belgium.be/eportal/index.htm |
| Belgium: Pharmaceutical Inspectorate | https://www.fagg-afmps.be/en |
| Croatia: Ministry of Health and Social Care | http://www.vlada.hr/en/naslovnica/o_vladi_rh/ministarstva/ministarstvo_zdravstva_i_socijalne_skrbi |
| Danish Medicines Agency | http://laegemiddelstyrelsen.dk/en |
| Denmark: Ministry of Food, Agriculture and Fisheries | http://www.fvm.dk/home.aspx?ID=14541 |
| Denmark: Ministry of Health | http://www.sum.dk/English.aspx |
| Denmark: Veterinary and Food Administration | https://www.foedevarestyrelsen.dk/english/Pages/default.aspx |
| Estonia: Ministry of Social Affairs | http://www.sm.ee/eng.html |
| Estonia: State Agency of Medicines | http://www.sam.ee/ |
| European Commission Consumer Affairs:  Medical Devices | http://ec.europa.eu/consumers/sectors/medical-devices/index_en.htm |
| European Commission Directorate General for Health & Consumers | http://ec.europa.eu/health/index_en.htm |
| European Commission Directorate General: Health Notice to Applicants | http://ec.europa.eu/health/documents/eudralex/index_en.htm |
| European Commission Directorate General: Medicinal Products for Veterinary Use | http://ec.europa.eu/health/human-use/index_en.htm |
| European Medicines Agency | http://www.ema.europa.eu/ema/index.jsp?curl=/pages/home/Home_Page.jsp&jsenabled=true |
| Finland: Ministry of Social Affairs and Health | http://www.stm.fi/en/frontpage |
| Finnish Food Safety Authority Evira | http://www.evira.fi/portal/en/ |
| Finnish Medicines Agency | http://www.fimea.fi/frontpage |
| France: National Agency for Veterinary Medicinal Products | https://www.anses.fr/en/content/french-agency-veterinary-medicinal-products |
| Germany: Federal Institute for Drugs and Medical Devices | http://www.bfarm.de/DE/Home/home_node.html |
| Germany: Ministry of Health | http://www.bmgesundheit.de/ |
| Greece: Hellenic Food Authority | http://www.efet.gr/ |
| Greece: Hellenic Ministry of Agriculture | http://www.minagric.gr/en/index.html |
| Greece: National Organization for Medicines | http://www.eof.gr/Welcome3_en.htm |
| Iceland: The Environment Agency | http://www.fisheries.is/management/institutes/the-environment-agency-of-iceland/ |
| Ireland: Agriculture and Food Development Authority | http://www.teagasc.ie/ |
| Ireland: Department of Health and Children | http://www.doh.ie/ |
| Ireland: Food Safety Authority | http://www.fsai.ie/ |
| Irish Medicines Board | http://www.imb.ie/ |
| Lithuania: Ministry of Health | http://www.sam.lt/go.php/News |
| Lithuania: State Medicines Control Agency | http://www.vvkt.lt/index.php?3327723903 |
| Malta: Ministry of Health, Elderly and Community Care | http://www.health.gov.mt/ |
| Netherlands: Ministry of Health, Welfare and Sport | http://english.minvws.nl/en/ |
| Norway: Ministry of Agriculture and Food | http://odin.dep.no/ld/engelsk/index-b-n-a.html |
| Norway: Ministry of Health and Care Services | http://www.regjeringen.no/en/dep/hod.html |
| Norway: Norwegian Board of Health Supervision | http://www.helsetilsynet.no/english.htm |
| Norway: Norwegian Medicines Agency | http://www.mattilsynet.no/portal/page?_pageid=54,40103&_dad=portal&_schema=PORTAL&language=english |
| Poland: Drug Institute | http://www.il.waw.pl/ |
| Slovak Republic: Ministry of Agriculture and Rural Development | http://www.mpsr.sk/en/?start |
| Slovenia: Ministry of Agriculture, Forestry and Food | http://www.gov.si/mkgp/slo/index.htm |
| Sweden: Medical Products Agency | https://lakemedelsverket.se/ |
| Sweden: Ministry for Rural Affairs | http://www.sweden.gov.se/sb/d/2064 |
| Sweden: National Food Administration | http://www.slv.se/en-gb/ |
| Switzerland: Federal Office of Public Health | http://www.bag.admin.ch/index.html?lang=en |
| Switzerland: Federal Veterinary Office | http://www.bfr.bund.de/en/swiss_federal_veterinary_office_sfvo-6324.html |
| UK: Department of Health | http://www.doh.gov.uk/ |
| UK: Food Standards Agency | http://www.food.gov.uk/ |
| UK: Health Protection Agency | http://www.hpa.org.uk/ |
| UK: Medicines and Healthcare Products Regulatory Agency | http://www.mhra.gov.uk/ |
| UK: National Institute for Biological Standards and Control | http://www.nibsc.ac.uk/ |
| UK: Veterinary Medicines Directorate | http://www.vmd.gov.uk/ |
| **Regulatory authority websites: Middle East** | |
| Israel: Ministry of Health | http://www.health.gov.il/english/ |
| Israel: Ministry of Industry, Trade and Labor | http://www.tamas.gov.il/NR/exeres/B0B48981-357D-446F-AFAC-91A358E93C87.htm |
| Jordan: Ministry of Health | http://www.moh.gov.jo/MOH/En/home.php |
| Lebanon: Ministry of Public Health | http://www.moph.gov.lb/Pages/Home.aspx |
| Saudi Arabia: Ministry of Health | http://www.moh.gov.sa/en/Pages/Default.aspx |
| United Arab Emirates: Ministry of Health | http://www.moh.gov.ae/en/Pages/default.aspx |
| United Arab Emirates: Federal Department of Pharmacies | http://www.moh.gov.ae/en/Pages/default.aspx |
| Yemen: Ministry of Public Health & Population | http://www.moh.gov.ye/ |
| **Regulatory authority websites: Africa** | |
| Botswana: Ministry of Health | http://www.moh.gov.bw/ |
| Egypt: Ministry of Agriculture and Land Reclamation | http://www.agr-egypt.gov.eg/En_Default.aspx |
| Ghana: Ministry of Health | http://www.moh-ghana.org/ |
| Ghana: Ministry of Food and Agriculture | http://mofa.gov.gh/ |
| Kenya: Ministry of Health | http://www.health.go.ke/ |
| Maldives: Ministry of Health | http://www.health.gov.mv/ |
| Mauritius: Ministry of Health & Quality of Life | http://health.gov.mu/ |
| Mauritius: Ministry of Agro Industry and Food Security | http://agriculture.gov.mu/ |
| Namibia: Ministry of Health and Social Services | http://www.healthnet.org.na/ |
| Namibia: Ministry of Fisheries and Marine Resources | http://www.mfmr.gov.na/ |
| South Africa: Department of Health | http://www.doh.gov.za/ |
| Swaziland: Ministry of Health and Social Welfare | http://www.gov.sz/index.php?option=com_content&view=article&id=267&Itemid=403 |
| Tanzania: Ministry of Health | http://www.tanzania.go.tz/government/health.htm |
| Uganda: Ministry of Health | http://www.health.go.ug/ |
| Zimbabwe: Ministry of Health and Child Welfare | http://www.mohcw.gov.zw/ |
| **Regulatory authority websites: America** | |
| Belize: Ministry of Health | http://www.health.gov.bz/www/ |
| Bolivia: Ministry of Health and Social Welfare | https://www.minsalud.gob.bo/ |
| Brazil: Fundacao Oswaldo Cruz | http://www.fiocruz.br/cgi/cgilua.exe/sys/start.htm?UserActiveTemplate=template_espanhol&tpl=home |
| Canada: Food Inspection Agency | http://www.inspection.gc.ca/english/toce.shtml |
| Canada: Health Products and Food Branch | http://www.hc-sc.gc.ca/ahc-asc/branch-dirgen/hpfb-dgpsa/index-eng.php |
| Guyana: Ministry of Health | http://www.health.gov.gy/ |
| Guyana: National Bureau of Standards | http://www.gnbsgy.org/ |
| Jamaica: Ministry of Health | http://www.moh.gov.jm/ |
| Mexico: Federal Commission for the Protection Against Sanitary Risks | http://www.cofepris.gob.mx/wb/cfp/ingles |
| Netherlands Antilles: Department of Public Health and Environmental Protection | http://www.sintmaartengov.org/government/VSA/Inspectorate%20Public%20Health,%20Social%20Services%20and%20Labor/Pages/default.aspx |
| St. Lucia: Ministry of Agriculture, Lands Forestry and Fisheries | http://www.maff.egov.lc/ |
| Trinidad & Tobago: Bureau of Standards | http://www.ttbs.org.tt/ |
| Trinidad and Tobago: Ministry of Health | http://www.health.gov.tt/ |
| **Regulatory authority websites: Multinational** | |
| World Health Organization | http://www.who.int/ |
| Pan-American Health Organization | http://www.paho.org/ |
| WHO Regional Office for Europe | http://www.who.dk/ |
| WHO Regional Office for Africa | http://www.afro.who.int/ |
| WHO Regional Office for the Eastern Mediterranean | http://www.emro.who.int/index.asp |
| WHO Regional Office for Southeast Asia | http://www.searo.who.int/ |
| WHO Regional Office for the Western Pacific | http://www.wpro.who.int/ |
| Food and Agriculture Organizations of the United Nations | http://www.fao.org/ |
| Codex Alimentarius | http://www.codexalimentarius.net/ |
| World Trade Organization | http://www.wto.org/ |
| **Other Sources** | |
| Crowdsourcing – medical focus | https://www.crowdflower.com/discovering-drug-side-effects-with-crowdsourcing/  http://www.sermo.com  http://www.medhelp.org  http://curetogether.com  https://www.upwork.com  https://fold.it/portal/  https://www.mturk.com/mturk/welcome  https://www.patientslikeme.com |
| Stanford links | http://crowdresearch.stanford.edu/w/index.php?title=Introducing_Crowd_Research_Initiative_and_Recap |
| **Government drug regulators (this list is key, however, see the WHO list for all countries or EMA for Europe) and related research agencies** | |
| Australia-Therapeutic Good Administration | http://www.tga.gov.au |
| Canada data | http://open.canada.ca/en |
| Canada ICES | http://www.ices.on.ca/About-ICES.aspx |
| European Commission | http://ec.europa.eu/growth/index_en.htm http://ec.europa.eu/health/human-use/advanced-therapies/index_en.htm |
| European Medicines Agency | http://www.ema.europa.eu/ema/index.jsp?curl=pages/regulation/document_listing/document_listing_000345.jsp -  http://www.ema.europa.eu/ema/index.jsp?curl=pages/medicines/general/general_content_000155.jsp |
| Finish Medicine Agency | http://www.fimea.fi/web/en/frontpage |
| France | http://www.afssaps.fr |
| Germany-Federal Institute for Drugs | http://www.bfarm.de/EN/Home/home_node.html;jsessionid=7CCFCA856FC813AC19DD69E86C4742CB.1_cid322 |
| Health Canada | http://www.hc-sc.gc.ca/dhp-mps/index-eng.php |
| NEHI | http://www.nehi.net |
| Netherland Medicines Evaluation Board | http://english.cbg-meb.nl |
| New Zealand | http://www.health.govt.nz |
| PHAC | http://www.phac-aspc.gc.ca/index-eng.php |
| StatsCan | http://www.statcan.gc.ca |
| Sweden | https://lakemedelsverket.se/english/ |
| US data | https://www.data.gov |
| US CDC | http://www.cdc.gov |
| US IOM | http://www.nationalacademies.org/hmd/ |
| US FDA | http://www.fda.gov/Drugs/GuidanceComplianceRegulatoryInformation/Surveillance/AdverseDrugEffects/ucm083765.htm |
| US NIH | https://www.nih.gov |
| US NIMH | http://www.nimh.nih.gov/ |
| UK Medicine and Health Products Regulator Agency | https://www.gov.uk/government/organisations/medicines-and-healthcare-products-regulatory-agency |
| WHO-search site, publications section and WHOLIS database | http://www.who-umc.org/DynPage.aspx?id=98080&mn1=7347&mn2=7252&mn3=7322&mn4=7324  http://www.who.int/en/  http://www.who.int/library/databases/en/  http://www.who.int/medicines/areas/quality_safety/regulation_legislation/list_mra_websites_nov2012.pdf  https://www.pda.org/scientific-and-regulatory-affairs/regulatory-resources/global-regulatory-authority-websites |
| **Pharma-related** | |
| FDABLE | http://www.fdable.com/basic_query/aers |
| EurdraVigiance | http://eudravigilance.ema.europa.eu/highres.htm |
| Social Media, Mobile, Wearable News and Views | http://www.scoop.it/t/pharmaguy-s-social-media-news-views/?tag=adverse+events |
| Innovative medicine initiative | http://www.imi.europa.eu |
| **Software** | |
| Open PHACTS | https://www.openphacts.org http://www.openphactsfoundation.org |
| DebugIT | http://www.debugit.com.au |
| Khresmoi | http://khresmoi.atosresearch.eu |
| EHR4CR | http://www.ehr4cr.eu |
| **Related article search results** | |
| **Clark Freifeld author search and related article** | Digital pharmacovigilance: The MedWatcher system for monitoring adverse events through automated processing of Internet social media and crowdsourcing (Thesis). Freifeld, Clark C., Ph.D., BOSTON UNIVERSITY, 2014, 148 pages; http://gradworks.umi.com/35/81/3581025.html  Freifeld CC, Chunara R, Mekaru SR, Chan EH, Kass-Hout T, et al. (2010) Participatory Epidemiology: Use of Mobile Phones for Community-Based Health Reporting. PLoS Med 7(12): e1000376. doi:10.1371/journal.pmed.1000376  Freifeld CC, Brownstein JS, Menone CM *et al*. [Digital drug safety surveillance: monitoring pharmaceutical products in Twitter](http://rd.springer.com/article/10.1007%2Fs40264-014-0155-x). *Drug Safety*2014; 37:343–350.  Guidance for Industry Internet/Social Media Platforms with Character Space Limitations— Presenting Risk and Benefit Information for Prescription Drugs and Medical Devices - http://www.fda.gov/downloads/drugs/guidancecomplianceregulatoryinformation/guidances/ucm401087.pdf  Inman WH: Attitudes to adverse drug-reaction reporting. Br J Clin Pharmacol 1996; 41:433-435  Lardon J, Abdellaoui R, Bellet F, Asfari H, Souvignet J, Texier N, Jaulent MC, Beyens MN, Burgun A, Bousquet C. Adverse Drug Reaction Identification and Extraction in Social Media: A Scoping Review J Med Internet Res 2015; 17(7):e171. <http://www.jmir.org/2015/7/e171/>  Lopez- Gonzalez E, Herdeiro MT, Figueiras A: Determinants of under-reporting of adverse drug reactions: a systematic review. Drug Saf 2009; 32:19-31; Hugman B. The fatal love of forms. Drug Saf 2011; 34 (8): 705-707.  R. Edwards and M. Lindquist: Social Media and Networks in Pharmacovigilance. Boon or Bane? Drug Saf 2011; 34 (4): 267-271  Manhattan Research 2011. Cybercitizen Health Europe v10.0. Manhattan Research 2009. Navigating the European eHealth Landscape.  Sai Moturu and Huan Liu. "Quantifying the Trustworthiness of Social Media Content", Journal of Distributed and Parallel Databases, Springer, Volume 29, January 4, 2011. DOI: 10.1007/s10619-010-7077-0. Geoffrey Barbier, and Huan Liu. Information Provenance in Social Media. SBP 2011: 276-283. Springer-Verlag Berlin, Heidelberg, ISBN: 978-3-642-19655-3  The Impact and Use of Social Media in Pharmacovigilance. https://www.sciformix.com/wp-content/uploads/Social_Media_in_PV_Whitepaper.pdf  See report WEBAE project (Web Adverse Events). http://www.imi.europa.eu/webfm_send/912 |

# Appendix 5. List of included studies

| **Article ID** | **Main documents (n=70)** |
| --- | --- |
| Abou Taam 2012[7] | Taam AM, Rossard C, Cantaloube L, Bouscaren N, Pochard L, Montastruc F et al. Analyze of internet narratives on patient websites before and after benfluorex withdrawal and media coverage. Fundam Clin Pharmacol. 2012;26:79-80. |
| Akay 2015[8] | Akay A, Dragomir A, Erlandsson B-E. Network-based modeling and intelligent data mining of social media for improving care. IEEE journal of biomedical and health informatics. 2015;19(1):210-218. |
| Alvaro 2015[9] | Alvaro N, Conway M, Doan S, Lofi C, Overington J, Collier N. Crowdsourcing Twitter annotations to identify first-hand experiences of prescription drug use. J Biomed Inform. 2015;58:280-287. |
| Benton 2011[10] | Benton A, Ungar L, Hill S, Hennessy S, Mao J, Chung A et al. Identifying potential adverse effects using the web: A new approach to medical hypothesis generation. J Biomed Inform. 2011;44(6):989-996. |
| Beusterien 2013[11] | Beusterien K, Tsay S, Gholizadeh S, Su Y. Real-world experience with colorectal cancer chemotherapies: patient web forum analysis. Ecancermedicalscience. 2013;7:361. |
| Bian 2012[12] | Bian J, Topaloglu U, Yu F. Towards large-scale twitter mining for drug-related adverse events. In: Proceedings of the 2012 international workshop on Smart health and wellbeing: 2012: ACM; 2012: 25-32. |
| Cameron 2014[13] | Cameron D, Sheth AP, Jaykumar N, Thirunarayan K, Anand G, Smith GA. A hybrid approach to finding relevant social media content for complex domain specific information needs. Web Semantics: Science, Services and Agents on the World Wide Web. 2014;29:39-52. |
| Carbonell 2015[14] | Carbonell P, Mayer MA, Bravo À. Exploring brand-name drug mentions on Twitter for pharmacovigilance. Stud Health Technol Inform. 2015;210:55-59. |
| Chary 2014[15] | Chary M, Park EH, McKenzie A, Sun J, Manini AF, Genes N. Signs & symptoms of dextromethorphan exposure from YouTube. PLoS One. 2014;9(2):e82452. |
| Chee 2009[16] | Chee BW, Berlin R, Schatz BR. Measuring population health using personal health messages. In: AMIA: 2009; 2009. |
| Chee 2011[17] | Chee BW, Berlin R, Schatz B. Predicting adverse drug events from personal health messages. In: AMIA Annu Symp Proc: 2011; 2011: 217-226. |
| Coloma 2015[18] | Coloma PM, Becker B, Sturkenboom MC, van Mulligen EM, Kors JA. What Can Social Media Networks Contribute To Medicines Safety Surveillance? In: Pharmacoepidemiol Drug Saf: 2015: Wiley-Blackwell 2015: 467-468. |
| Correia 2016[19] | Correia RB, Li L, Rocha LM. Monitoring potential drug interactions and reactions via network analysis of instagram user timelines. In: Pacific Symposium on Biocomputing Pacific Symposium on Biocomputing: 2016: NIH Public Access; 2016: 492. |
| Elhadad 2014[20] | Elhadad N, Zhang S, Driscoll P, Brody S. Characterizing the sublanguage of online breast cancer forums for medications, symptoms, and emotions. In: Proc AMIA Annual Fall Symposium: 2014; 2014. |
| Freifeld 2014a[21] | Freifeld CC, Brownstein JS, Menone CM, Bao W, Filice R, Kass-Hout T et al. Digital drug safety surveillance: monitoring pharmaceutical products in twitter. Drug Saf. 2014;37(5):343-350. |
| Freifeld 2014b[22] | Freifeld CC. Digital Pharmacovigilance: the medwatcher system for monitoring adverse events through automated processing of internet social media and crowdsourcing. 2014. |
| Ginn 2014[23] | Ginn R, Pimpalkhute P, Nikfarjam A, Patki A, O’Connor K, Sarker A et al. Mining Twitter for adverse drug reaction mentions: a corpus and classification benchmark. In: Proceedings of the fourth workshop on building and evaluating resources for health and biomedical text processing: 2014: Citeseer; 2014. |
| Gupta 2014[24] | Gupta S, MacLean DL, Heer J, Manning CD. Induced lexico-syntactic patterns improve information extraction from online medical forums. J Am Med Inform Assoc. 2014;21(5):902-909. |
| Hadzi-Puric 2012[25] | Hadzi-Puric J, Grmusa J. Automatic drug adverse reaction discovery from parenting websites using disproportionality methods. In: Proceedings of the 2012 International Conference on Advances in Social Networks Analysis and Mining (ASONAM 2012): 2012: IEEE Computer Society; 2012: 792-797. |
| Hanson 2013a[26] | Hanson CL, Burton SH, Giraud-Carrier C, West JH, Barnes MD, Hansen B. Tweaking and tweeting: exploring Twitter for nonmedical use of a psychostimulant drug (Adderall) among college students. J Med Internet Res. 2013;15(4):e62. |
| Hanson 2013b[27] | Hanson CL, Cannon B, Burton S, Giraud-Carrier C. An exploration of social circles and prescription drug abuse through Twitter. J Med Internet Res. 2013;15(9):e189. |
| Hughes 2011[28] | Hughes S, Cohen D. Can online consumers contribute to drug knowledge? A mixed-methods comparison of consumer-generated and professionally controlled psychotropic medication information on the internet. J Med Internet Res. 2011;13(3):e53. |
| Jimeno-Yepes 2014[29] | Jimeno-Yepes A, MacKinlay A, Han B, Chen Q. Identifying Diseases, Drugs, and Symptoms in Twitter. Stud Health Technol Inform. 2014;216:643-647. |
| Johnson 2013[30] | Johnson HK, Nancy. A side effect of social media. What can twitter tell us about adverse drug reactions. In: UKMI 39th Professional Development Seminar: 2013; 2013. |
| Karimi 2015a[31] | Karimi S, Metke-Jimenez A, Kemp M, Wang C. Cadec: A corpus of adverse drug event annotations. J Biomed Inform. 2015;55:73-81. |
| Karimi 2015b[32] | Karimi S, Metke-Jimenez A, Nguyen A. CADEminer: A System for Mining Consumer Reports on Adverse Drug Side Effects. In: Proceedings of the Eighth Workshop on Exploiting Semantic Annotations in Information Retrieval: 2015: ACM; 2015: 47-50. |
| Kmetz 2011[33] | Kmetz J. Pharmaceutical industry special report: Adverse Event Reporting in Social Media. In.: Visible; 2011. |
| Leaman 2010[34] | Leaman R, Wojtulewicz L, Sullivan R, Skariah A, Yang J, Gonzalez G. Towards internet-age pharmacovigilance: extracting adverse drug reactions from user posts to health-related social networks. In: Proceedings of the 2010 workshop on biomedical natural language processing: 2010: Association for Computational Linguistics; 2010: 117-125. |
| Liu 2011[35] | Liu J, Li A, Seneff S. Automatic drug side effect discovery from online patient-submitted reviews: Focus on statin drugs. In: Proceedings of First International Conference on Advances in Information Mining and Management (IMMM): 2011; Barcelona, Spain; 2011: 23-29. |
| Liu 2014[36] | Liu X, Liu J, Chen H. Identifying adverse drug events from health social media: a case study on heart disease discussion forums. In: International Conference on Smart Health: 2014: Springer; 2014: 25-36. |
| Liu 2015a[37] | Liu X, Chen H. A research framework for pharmacovigilance in health social media: Identification and evaluation of patient adverse drug event reports. J Biomed Inform. 2015;58:268-279. |
| Liu 2015b[38] | Liu XC, Hsinchun. Identifying Adverse Drug Events from Health Social Media Using Distant Supervision. In: INFORMS Conference on Information Systens and Technology. Philadelphia; 2015. |
| Mao 2013[39] | Mao JJ, Chung A, Benton A, Hill S, Ungar L, Leonard CE et al. Online discussion of drug side effects and discontinuation among breast cancer survivors. Pharmacoepidemiol Drug Saf. 2013;22(3):256-262. |
| McGinley 2015[40] | McGinley M, Alinia H, Kuo S, Huang KE, Feldman SR. Patient perspectives on low level light therapy and laser therapies for rosacea-associated persistent facial redness. Dermatol Online J. 2015;21(2). |
| Medawar 2012[41] | Medawar C, Herxheimer A, Bell A, Jofre S. Paroxetine, Panorama and user reporting of ADRs: Consumer intelligence matters in clinical practice and post‐marketing drug surveillance. Int J Risk Saf Med. 2002;15(3, 4):161-169. |
| Metke-Jimenez 2014[42] | Metke-Jimenez A, Karimi S, Paris C. Evaluation of text-processing algorithms for adverse drug event extraction from social media. In: Proceedings of the first international workshop on Social media retrieval and analysis: 2014: ACM; 2014: 15-20. |
| Metke-Jimenez 2015[43] | Metke-Jimenez A, Karimi S. Concept extraction to identify adverse drug reactions in medical forums: A comparison of algorithms. arXiv preprint arXiv:150406936. 2015. |
| Nadarajah 2015[44] | Nadarajah S. Monitoring Adverse Events in Pharma’s Patient Support Programs. In.: IMS Health; 2015. |
| Nikfarjam 2011[45] | Nikfarjam A, Gonzalez GH. Pattern mining for extraction of mentions of adverse drug reactions from user comments. In: AMIA Annu Symp Proc: 2011; 2011: 1019-1026. |
| Nikfarjam 2015[46] | Nikfarjam A, Sarker A, O’Connor K, Ginn R, Gonzalez G. Pharmacovigilance from social media: mining adverse drug reaction mentions using sequence labeling with word embedding cluster features. J Am Med Inform Assoc. 2015;22(3):671-681. |
| Oleson 2013[47] | Dole Oleson. Discovering Drug Side Effects with Crowdsourcing. 2013. https://www.crowdflower.com/discovering-drug-side-effects-with-crowedsourcing/. |
| Pages 2014[48] | Pages A, Bondon-Guitton E, Montastruc JL, Bagheri H. Undesirable effects related to oral antineoplastic drugs: comparison between patients’ internet narratives and a national pharmacovigilance database. Drug Saf. 2014;37(8):629-637. |
| Patki 2014[49] | Patki A, Sarker A, Pimpalkhute P, Nikfarjam A, Ginn R, O’Connor K et al. Mining adverse drug reaction signals from social media: going beyond extraction. Proceedings of BioLinkSig. 2014;2014:1-8. |
| Pimpalkhute 2014[50] | Pimpalkhute P, Patki A, Nikfarjam A. Phonetic spelling filter for keyword selection in drug mention mining from social media. 2014. |
| Powell 2016[51] | Powell GE, Seifert HA, Reblin T, Burstein PJ, Blowers J, Menius JA et al. Social media listening for routine post-marketing safety surveillance. Drug Saf. 2016;39(5):443-454. |
| Risson 2015[52] | Risson V, Saini D, Bonzani I, Huisman A, Olson M. Validation of Social Media Analysis for Outcomes Research: Identification of Drivers of Switches between oral and Injectable Therapies for Multiple Sclerosis. Value Health. 2015;18(7):A729. |
| Rizo 2011[53] | Rizo C, Deshpande A, Ing A, Seeman N. A rapid, Web-based method for obtaining patient views on effects and side-effects of antidepressants. J Affect Disord. 2011;130(1):290-293. |
| Samparthkuman 2014[54] | Sampathkumar H, Chen X-w, Luo B. Mining adverse drug reactions from online healthcare forums using hidden Markov model. BMC Med Inform Decis Mak. 2014;14(1):1. |
| Sarker 2015[55] | Sarker A, Gonzalez G. Portable automatic text classification for adverse drug reaction detection via multi-corpus training. J Biomed Inform. 2015;53:196-207. |
| Sarker 2016a[56] | Sarker A, Nikfarjam A, Gonzalez G. Social media mining shared task workshop. In: Proceedings of the Pacific Symposium on Biocomputing: 2016; 2016. |
| Sarker 2016b[57] | Sarker A, O’Connor K, Ginn R, Scotch M, Smith K, Malone D et al. Social media mining for toxicovigilance: automatic monitoring of prescription medication abuse from Twitter. Drug Saf. 2016;39(3):231-240. |
| Sarrazin 2014[58] | Sarrazin MSV, Cram P, Mazur A, Ward M, Reisinger HS. Patient perspectives of dabigatran: analysis of online discussion forums. The Patient-Patient-Centered Outcomes Research. 2014;7(1):47-54. |
| Scanfeld 2010[59] | Scanfeld D, Scanfeld V, Larson EL. Dissemination of health information through social networks: Twitter and antibiotics. Am J Infect Control. 2010;38(3):182-188. |
| Schröder 2007[60] | Schröder S, Zöllner YF, Schaefer M. Drug related problems with Antiparkinsonian agents: consumer Internet reports versus published data. Pharmacoepidemiol Drug Saf. 2007;16(10):1161-1166. |
| Segura-Bedmar 2014[61] | Segura-Bedmar I, Revert R, Martínez P. Detecting drugs and adverse events from Spanish health social media streams. In: Proceedings of the 5th International Workshop on Health Text Mining and Information Analysis (Louhi)@ EACL: 2014; 2014: 106-115. |
| Segura-Bedmar 2015[62] | Segura-Bedmar I, Martínez P, Revert R, Moreno-Schneider J. Exploring Spanish health social media for detecting drug effects. BMC Med Inform Decis Mak. 2015;15(2):1. |
| Shutler 2015[63] | Shutler L, Nelson LS, Portelli I, Blachford C, Perrone J. Drug use in the Twittersphere: a qualitative contextual analysis of tweets about prescription drugs. J Addict Dis. 2015;34(4):303-310. |
| Topaz 2016[64] | Topaz M, Lai K, Dhopeshwarkar N, Seger DL, Sa’adon R, Goss F et al. Clinicians’ Reports in Electronic Health Records Versus Patients’ Concerns in Social Media: A Pilot Study of Adverse Drug Reactions of Aspirin and Atorvastatin. Drug Saf. 2016;39(3):241-250. |
| White 2013[65] | White RW, Tatonetti NP, Shah NH, Altman RB, Horvitz E. Web-scale pharmacovigilance: listening to signals from the crowd. J Am Med Inform Assoc. 2013;20(3):404-408. |
| White 2014[66] | White RW, Harpaz R, Shah NH, DuMouchel W, Horvitz E. Toward enhanced pharmacovigilance using patient-generated data on the internet. Clin Pharmacol Ther. 2014;96(2):239. |
| Whitman 2014[67] | Whitman CB, Reid MW, Arnold C, Patel H, Ursos L, Sa'adon R et al. Balancing opioid-induced gastrointestinal side effects with pain management: Insights from the online community. Journal of opioid management. 2014;11(5):383-391. |
| Wu 2013[68] | Wu H, Fang H, Stanhope S. Exploiting online discussions to discover unrecognized drug side effects. Methods Inf Med. 2013;52(2):152-159. |
| Yang 2012a[69] | Yang CC, Jiang L, Yang H, Tang X. Detecting signals of adverse drug reactions from health consumer contributed content in social media. In: Proceedings of ACM SIGKDD Workshop on Health Informatics: 2012; 2012. |
| Yang 2012b[70] | Yang CC, Yang H, Jiang L, Zhang M. Social media mining for drug safety signal detection. In: Proceedings of the 2012 international workshop on Smart health and wellbeing: 2012: ACM; 2012: 33-40. |
| Yang 2013[71] | Yang H, Yang CC. Harnessing social media for drug-drug interactions detection. In: Healthcare Informatics (ICHI), 2013 IEEE International Conference on: 2013: IEEE; 2013: 22-29. |
| Yang 2015a[72] | Yang M, Kiang M, Shang W. Filtering big data from social media–Building an early warning system for adverse drug reactions. J Biomed Inform. 2015;54:230-240. |
| Yang 2015b[73] | Yang CC, Yang H. Exploiting Social Media with Tensor Decomposition for Pharmacovigilance. In: 2015 IEEE International Conference on Data Mining Workshop (ICDMW): 2015: IEEE; 2015: 188-195. |
| Yates 2013a[74] | Yates A, Goharian N, Frieder O. Extracting adverse drug reactions from forum posts and linking them to drugs. In: Proceedings of the 2013 ACM SIGIR Workshop on Health Search and Discovery: 2013; 2013. |
| Yates 2013b[75] | Yates A, Goharian N. ADRTrace: Detecting Expected and Unexpected Adverse Drug Reactions from User Reviews on Social Media Sites. In: Advances in Information Retrieval: 35th European Conference on IR Research. edn. Edited by Serdyukov P, Braslavski P, Kuznetsov SO, Kamps J, Rüger S, Agichtein E, Segalovich I, Yilmaz E. Berlin, Heidelberg: Springer Berlin Heidelberg; 2013: 816-819. |
| Yom-Tov 2013[76] | Yom-Tov E, Gabrilovich E. Postmarket drug surveillance without trial costs: discovery of adverse drug reactions through large-scale analysis of web search queries. J Med Internet Res. 2013;15(6):e124. |
| **Article ID** | **Companion documents (n=7)** |
| Karimi 2015a [CR: Karimi 2011][77] | Karimi S, Kim S, Cavedon L. Drug side-effects: What do patient forums reveal. In: The second international workshop on Web science and information exchange in the medical Web: 2011: ACM; 2011: 10-11. |
| Liu 2010 [CR: Li 2011][78] | Li YA. Medical data mining: Improving information accessibility using online patient drug reviews. Massachusetts Institute of Technology; 2011. |
| Liu 2015a [CR: Liu 2013][79] | Liu X, Chen H. AZDrugMiner: an information extraction system for mining patient-reported adverse drug events in online patient forums. In: International Conference on Smart Health: 2013: Springer; 2013: 134-150. |
| Liu 2015a [CR: Liu 2015c][80] | Liu X, Chen H. Identifying adverse drug events from patient social media: A case study for diabetes. IEEE Intelligent Systems. 2015;30(3):44-51. |
| Ginn 2014 [CR: O’Connor 2014][81] | O’Connor K, Pimpalkhute P, Nikfarjam A, Ginn R, Smith KL, Gonzalez G. Pharmacovigilance on Twitter? Mining Tweets for adverse drug reactions. In: AMIA Annual Symposium Proceedings: 2014: American Medical Informatics Association; 2014: 924. |
| Segura-Bedmar 2015 [CR: Segura-Bedmar 2014][82] | Segura-Bedmar I, De La Peña S, Martınez P. Extracting drug indications and adverse drug reactions from Spanish health social media. In: Proceedings of BioNLP: 2014; 2014: 98-106. |
| Metke-Jimenez 2014 [CR: Wang 2014][83] | Wang C, Karimi S. Differences between social media and regulatory databases in adverse drug reaction discovery. In: Proceedings of the first international workshop on Social media retrieval and analysis: 2014: ACM; 2014: 13-14. |

# Appendix 6. Social media data processing pipeline

| **Data processing (n=70)** | | **Count (%)** |
| --- | --- | --- |
| Type of machine learning used to process social media data | Supervised learning | 15 (21.4%) |
|  | Rule-based learning | 6 (8.6%) |
|  | Semi-supervised learning | 5 (7.1%) |
|  | Unsupervised machine learning | 4 (5.7%) |
|  | Machine learning unspecified | 3 (4.3%) |
|  | Distant supervised learning | 2 (2.9%) |
|  | Statistical learning | 2 (2.9%) |
|  | Supervised and Deep learning | 1 (1.4%) |
|  | None applied | 16 (22.9%) |
|  | Not applicable (no system, descriptive paper) | 16 (22.9%) |
| Pre-processing of social media data | Automated | 32 (45.7%) |
|  | Not reported | 22 (31.4%) |
|  | Not applicable (no system, descriptive paper) | 16 (22.9%) |
| De-identification of social media data | Automated | 5 (7.1%) |
|  | Semi-automated | 1 (1.4%) |
|  | Did not collect identifiable information | 5 (7.1%) |
|  | Not reported | 43 (61.4%) |
|  | Not applicable (no system, descriptive paper) | 16 (22.9%) |
| De-duplication of social media data | Automated | 5 (7.1%) |
|  | Manual | 1 (1.4%) |
|  | Not reported | 48 (68.6%) |
|  | Not applicable (no system, descriptive paper) | 16 (22.9%) |
| Concept identification in social media data* | Dictionary or lexicon-based approach | 30 (42.9 %) |
|  | Supervised classifier | 6 (8.6%) |
|  | Lexicon-based and supervised classifier | 2 (2.9%) |
|  | Rule-based phrase extraction | 2 (2.9%) |
|  | Sentiment analysis | 2 (2.9%) |
|  | Statistical model | 2 (2.9%) |
|  | Manual | 6 (8.6%) |
|  | Not reported | 5 (7.1 %) |
|  | Not applicable (no system, descriptive paper) | 16 (22.9%) |
| Concept normalization of ***drug names*** in social media data* | Dictionary or lexicon-based approach | 19 (27.1%) |
|  | Statistical model | 1 (1.4%) |
|  | Manual | 2 (2.9%) |
|  | Not reported | 33 (47.1%) |
|  | Not applicable (no system, descriptive paper) | 16 (22.9%) |
| Concept normalization *of* ***adverse events*** in social media data* | Dictionary or lexicon-based approach | 33 (47.1 %) |
|  | Statistical model | 2 (2.9%) |
|  | Manual | 5 (7.1%) |
|  | Not reported | 15 (21.4%) |
|  | Not applicable (no system, descriptive paper) | 16 (22.9%) |
| Relation extraction of drug and medical event pairs in social media data | Rule-based or statistical association mining | 16 (22.9%) |
|  | Supervised classifier | 15 (21.4%) |
|  | Dictionary or lexicon-based approach | 4 (5.7%) |
|  | Sentiment analysis | 2 (2.9%) |
|  | Manual | 4 (5.7%) |
|  | Not reported | 13 (18.6%) |
|  | Not applicable (no system, descriptive paper) | 16 (22.9%) |

*Note: Each document could contribute to more than one category.

# Appendix 7: Pre-existing social media listening platforms for drug safety surveillance

| **Article ID** | **Platform** | **Availability/Developer** |
| --- | --- | --- |
| Carbonell 2015[14] | BeFree System (Bio-Entity Finder & RElation Extraction) | Integrative Bioinformatics group |
| Freifeld 2014b[22] | MedWatcher Social | US Food and Drug Administration (FDA) |
| Kmetz 2011[33] | Visible Intelligence social media monitoring and analytics platform | Commercial service provided Cision |
| Nadarajah 2015[44] | AETracker | Commercial service provided by IMS health |
| Rizo 2011[53] | OpenCalais | Commercially available by Thomson Reuters |
| Segura-Bedmar 2014[61, 82] | MeaningCloud | Commercially available by MeaningCloud LLC, Sngular company |
| Topaz 2016, Whitman 2014[64, 67] | Treato | Commercially available by Treato Ltd. |
| Also, WEB-RADR Social Media under development jointly by Innovative Medicines Initiative (IMI) and European Union regulators | | |

# Appendix 8. Utility of social media data for pharmacovigilance

| **Article ID** | **Type of event** | **Social media data validated with** | **Validation measure** | **Author conclusion** |
| --- | --- | --- | --- | --- |
| ***Social media data vs. spontaneous reporting systems (n=10)*** | | | | |
| Freifeld et al., 2014[21] | Adverse Events | FDA Adverse Event Reporting System | Correlation | + |
| Johnson et al., 2013[30] | Adverse Events | UK Yellow Card | Frequencies | + |
| Kmetz et al., 2011[33] | Adverse Events | FDA’s Four Adverse Event Reporting Criteria | Frequencies | - |
| Leaman et al., 2010[34] | Adverse Events | FDA online drug library | Frequencies | + |
| Medawar et al., 2002[41] | Adverse Events | UK Yellow Card | Frequencies | + |
| Oleson et al., 2013[47] | Adverse Events | FDA Adverse Event Reporting System | Frequencies | + |
| Pages et al., 2014[48] | Adverse Events | French Pharmacovigilance Database (FPVD) | Frequencies | + |
| Powell et al., 2016[51] | Adverse Events | FDA Adverse Event Reporting System | Frequencies | + |
| Yom-Tov et al., 2013[76] | Adverse Events | FDA Adverse Event Reporting System, SIDER | Correlation | + |
| Yang et al., 2015[73] | Timing of adverse events | FDAs official alert or labeling revision time | Detection time | + |
| ***Social media data vs. published data (n=6)*** | | | | |
| Akay et al., 2015[8] | Adverse Events | Published data | Word frequency | + |
| Coloma et al., 2015[18] | Adverse Events | Previously known safety signals | Frequencies | + |
| Liu et al., 2010[35, 78] | Adverse Events | Published data | Frequencies | + |
| Mao et al., 2013[39] | Adverse events | Published clinical and observational data | Frequencies | + |
| Schroder et al., 2007[60] | Adverse Events | Published data | Frequencies | + |
| Sarrazin et al., 2014[58] | Adverse Events | Published clinical trial data | Qualitative | + |
| ***Social media data vs. drug withdrawn from the market(n=1)*** | | | | |
| Chee et al., 2015[17] | Adverse Events | Drugs withdrawn from the market | Weighted score | + |
| ***Social media data vs. expert confirmed cases (n=1)*** | | | | |
| Nadarajah et al., 2015[44] | Adverse Events | Confirmed by pharmacovigilance experts | Frequencies | - |
| ***Social media data vs. administrative database (n=1)*** | | | | |
| Topaz et al., 2016[64] | Adverse Events | Electronic health records | Frequencies | + |

*Note: None of the documents investigated utility or validity of social media data for pharmacovigilance as their primary objective and did not include a comprehensive comparison.*

*Symbols: + means agreement/consistent with comparator, – means not consistent with the comparator*

# Appendix 9. Pre-processing methods and results

| Article ID | Pre-processing description | Validity & reliability results |
| --- | --- | --- |
| Akay 2015[8] | Data were tokenized and stop words were filtered to ensure a uniform set of variables that can be measured. | None Provided. |
| Alvaro 2015[9] | Tweets were filtered for mention of target drugs and extraneous information (e.g. images, URLs,) was removed. | None Provided. |
| Benton 2011[10] | Data were filtered to remove extraneous information (e.g. images, URLs,) and tokenized. | None Provided. |
| Bian 2012[12] | Data were filtered for 5 cancer drugs using the drug name and its synonyms. Retweets and non-English tweets were removed as well as extraneous information was removed. | None Provided. |
| Chary 2014[15] | Removed English, French, and Spanish stop words as defined in Python’s NLTK (Natural Language Toolkit) and proprietary custom list. All characters that could not be encoded in ASCII and all non-alphanumeric characters were removed. | None Provided. |
| Chee 2009[16] | Texts were parsed to extract just the textual information and to remove noise. | None Provided. |
| Correia 2016[19] | Uppercase characters were converted to lowercase, and hashtag terms were treated like all other harvested texts. | None Provided. |
| Elhadad 2014[20] | Data were tokenized, converted to lowercase and stemmed. Images, URLs, and stop words were removed. | None Provided. |
| Freifeld 2014a[21] | Data were tokenized. | None Provided. |
| Ginn 2014[23, 81] | Remove extraneous information (e.g. images, URLs,) | None Provided. |
| Gupta 2014[24] | Stanford CoreNLP toolkit used to tokenize texts and tagged for their part-of-speech (POS) and lemmatized (i.e., canonical form). All texts are also converted into lowercase. | None Provided. |
| Hadzi-Puric 2012[25] | Data were tagged for part-of-speech using the TnT tagger for Serbian. Upper and lower case, affix, punctuation, inflectional endings and sorting were removed. | None Provided. |
| Jimeno-Yepes 2015[29] | Removed non-English tweets using LANGID.PY. | None Provided. |
| Leaman 2010[34] | Data were tokenized and tagged for part of-speech using the Hepple tagger. Stop words were removed and tokens were stemmed using the Snowball implementation of the Porter2 stemmer. | None Provided. |
| Liu 2011[35, 78] | Data were filtered for those indexed as “side_effects” in each review entry and all common stop words were removed. | None Provided. |
| Liu 2014[36] | Data were tokenized. | None Provided. |
| Liu 2015a[37, 79, 80] | Data were tokenized and extraneous information (e.g. images, URLs,) was removed. | None Provided. |
| Liu 2015b[38] | Data were tokenized and tagged for their part-of-speech and parsed. | None Provided. |
| Metke-Jimenez 2014[42] | Data were tokenized and filtering (tokens discarded or modified). | None Provided. |
| Metke-Jimenez 2015[43] | Data were tokenized. | None Provided. |
| Nikfarjam 2011[45] | Data were tokenized, stop words were removed and tagged for their part of speech. | None Provided. |
| Nikfarjam 2015[46] | Data were spell checked using Apache Lucene spell checker library and lemmatizatized using the Dragon toolkit lemmatizer. | None Provided. |
| Patki 2014[49] | Data were converted to lowercase and stemmed using the Porter stemmer. | None Provided. |
| Sampathkumar 2014[54] | Data were parsed to extract unique thread names and associated messages, HTML tags removed, converted to lowercase and filtered to remove extraneous information. Data were then tokenized and filtered of common stop words. | None Provided. |
| Sarker 2015[55] | Texts were tokenized, converted to lowercase and stemmed using Porter stemmer. Texts were then parsed using Twitter Part-Of-Speech (POS) Tagger and the Stanford parser and extraneous information (e.g. images, URLs,) was removed. | None Provided. |
| Sarker 2016b[57] | Tweets were stemmed using the Porter stemmer, converted to lowercase, tokenized and tagged for their part-of-speech. | None Provided. |
| Wu 2013[68] | Data were stemmed using Porter Stemmer. | None Provided. |
| Yang 2012a[69] | Data were tokenized and punctuations and stop words were removed. | None Provided. |
| Yang 2012b[70] | Data were tokenized and punctuations and stop words were removed. | None Provided. |
| Yang 2013[71] | Punctuations and stop words were removed. | None Provided. |
| Yang 2015a[72] | Punctuation, numbers, non-alphabet characters, and stop words were removed and words were stemmed. | None Provided. |
| Yates 2013a[74] | Data were tokenized using Punkt sentence tokenizer. | None Provided. |

# Appendix 10. De-identification methods and results

| **Article ID** | **De-identification (i.e., removal of personally identifiable information) method** | **Validity & reliability results** |
| --- | --- | --- |
| Benton 2011[10] | CRF-based de-identification of data. | Precision: 67.4%, Recall: 98.1%, F-measure: 79.9%. |
| Liu 2014[36] | Removed URLs, telephone numbers, SSN and other identifiable information. | None provided |
| Liu 2015a[37, 79, 80] | Removed identifiable information such as email addresses, social security numbers, and phone numbers from the text. | None provided |
| Sarker 2015[55] | Removed references to Twitter user names. | None provided |
| Sarker 2016a[56] | Removed usernames and hyperlinks. | None provided |
| Yom-Tov 2013[76] | The search logs were anonymized by scrambling actual user identifiers. | None provided |

# Appendix 11. De-duplication methods and results

| **Article ID** | **De-duplication (i.e., removal of duplicate posts) method** | **Validity & reliability results** |
| --- | --- | --- |
| Freifeld 2014b[22] | Duplicates identified using a sliding window of posts in chornological order (approximately one day), and compares the text of each subsequent post to all posts in the window. A built-in string comparison algorithm in PHP was subsequently used to compute a similarity percentage (set at 80%) for any two posts. | None provided |
| Jimeno-Yepes 2015[29] | Not reported. | None provided |
| Powell 2016[51] | Literal duplicates were removed using verbatim matches. Fuzzy matches were determined using a rule-based approach developed based on a Bloom filter. | None provided |
| Topaz 2016[64] | Duplicate documents from the same users were removed. | None provided |
| White 2013[65] | Anonymous unique identifiers were used to track search log (unable to detect multiple users from the same machine). | None provided |
| White 2014[66] | Anonymous unique identifiers were used to track search log (unable to detect multiple users from the same machine). | None provided |

# Appendix 12. Concept identification methods and results

| **Article ID** | **Method for concept identification** | | | | **Validity & reliability results** | | |
| --- | --- | --- | --- | --- | --- | --- | --- |
|  | **Approach type** | **Description** | **Tool or software used** | **Tool accessibility** | **Drug concepts** | **Adverse event concepts** | **Overall** |
| ***Dictionary or lexicon-based approach (n=30)*** | | | | | | | |
| Benton 2011[10] | Lexicon-based | Stemmed words automatically matched to a lexicon that was generated using MedicineNet, FAERS database and CHV. | Dictionary derived from MedicineNet, FAERS and CHV | Open-source | None provided | None provided | None provided |
| Bian 2012[12] | Dictionary | Used Metamap. | MetaMap | Open-access | None provided | None provided | None provided |
| Carbonell 2015[14] | Dictionary with fuzzy matching | Mined using BeFree System, a text mining tool for information extraction. | BeFree System | Unclear | None provided | None provided | None provided |
| Chee 2011[17] | Lexicon-based | Specialized lexicons used. | Proprietary tool | Proprietary | None provided | None provided | None provided |
| Correia 2016[19] | Lexicon-based | Timeline posts were tagged with terms from a symptom dictionary that was extracted from BICEPP by collecting all entries defined as an adverse effect. | Proprietary tool | Proprietary | None provided | None provided | None provided |
| Freifeld 2014a[21] | Dictionary | A tree-based dictionary-matching algorithm to identify both product and symptom mentions was used. Two separate product and symptom dictionaries were superimposed into a single tree for matching both product and symptom concepts at once. | Proprietary tool | Proprietary | None provided | P: 72%, R: 86%, F: 78% | None provided |
| Freifeld 2014b[22] | Lexicon-based | Lexicon-based approach using the HealthMap tree-based dictionary matching algorithm to identify both product and symptom mentions. | Proprietary system | Proprietary | None provided | P: 77%, R: 88%, F: 82% | None provided |
| Gupta 2014[24] | Dictionary | Extract symptom and conditions and drugs and treatments terms by matching to an automatically compiled dictionary, and inducing lexico-syntactic word patterns. | Proprietary dictionary | Proprietary | Asthma forum P: 86.9%, R: 58.7%, F: 70.0% ENT forum P: 82.8, R: 44.8%, F: 58.2% | Asthma forum P: 78.1%, R: 75.6%, F: 76.8% ENT forum P: 71.7%, R: 61.5%, F: 66.2% | None provided |
| Hadzi-Puric 2012[25] | Dictionary | MetaMap was used. | MetaMap | Open-access | None provided | P: 75.3%, R: 64.7%, F: 69.6% | None provided |
| Leaman 2010[34] | Lexicon-based | Concepts identified using the UMLS Metathesaurus, SIDER, and COSTART vocabulary | Not reported | Not applicable | None provided | P: 78.3%, Recall 69.9%, F-measure 73.9% | None provided |
| Liu 2014[36] | Lexicon-based | Lexicon-based approach to extract mentions of drugs and medical events using MetaMap. | MetaMap | Open access | P: 94.29%, R: 81.32%, F: 87.33% | P: 79.30%, R: 73.25%, F: 76.16% | None provided |
| Liu 2015a[37, 79, 80] | Lexicon-based | Lexicon-based approach to extract mentions of drugs and medical events using MetaMap and CHV. | UMLS-MetaMap, CHV | Open-access | American Diabetes Association P: 93.0%, R:91.7%, F:92.3% Diabetes Forums P: 92.5%, R:87.1%, F:89.7% Diabetes Forum P: 91.4%, R:86.4%, F:88.8% MedHelp (heart disease) P: 94.3%, R:81.3%, F:87.3% | American Diabetes Association P: 87.3%, R:80.3%, F: 83.6% Diabetes Forums P: 86.5%, R:78.7%, F: 82.5% Diabetes Forum P: 85.4%, R:76.5%, F: 80.7% MedHelp (heart disease) P: 79.3%, R:73.5%, F: 76.3% | None provided |
| Liu 2015b[38] | Lexicon-based | Lexicon-based approach to extract mentions of drug and medical concepts using MetaMap. | UMLS-MetaMap | Open-access | None provided | None provided | None provided |
| Metke-Jimenez 2014[42] | Lexicon-based | All of the controlled vocabularies used were linked to UMLS semantic type. | UMLS | open-access | Strict match P: 25.4%, R: 76.6%, F: 38.2% Relaxed match P: 28.2%, R: 83.9%, F: 42.2% | Strict match P: 46.3%, R: 43.8%, F: 45.0% Relaxed match P: 70.9%, R: 64.9%, F: 67.8% | None provided |
| Powell 2016[51] | Lexicon-based | Concepts identified with MedDRA | Natural language processing (NLP) software, MedDRA | Proprietary | None provided | None provided | None provided |
| Sampathkumar 2014[54] | Lexicon-based | Lexicon-based Named Entity Recognition. | Proprietary system | Proprietary | None provided | None provided | None provided |
| Sarker 2015[55] | Lexicon-based, dependency parses | Lexicon-based approach using UMLS-MetaMap. | UMLS-MetaMap, Stanford Parser | Open-access | None provided | None provided | None provided |
| Sarker 2016b[57] | Lexicon-based | Identified terms from a lexicon of drug abuse keywords | Proprietary system | Proprietary | None provided | None provided | None provided |
| Segura-Bedmar 2014[61, 82] | Lexicon-based | Lexicon-based approach using a researcher developed drug dictionary compiled from CIMA, ATC system and Vademecum and adverse event dictionary compiled from MedDRA, which was integrated into MeaningCloud and GATE gazetteers for named entity recognition. | MeaningCloud with GATE (gazetteers) plug-in | Fee-based | P: 86%, R: 81%, F: 84% | P: 63%, R: 51%, F: 57% |  |
| Segura-Bedmar 2015[62] | Lexicon-based | Lexicon-based Named Entity Recognition using GATE platform based on the MeaningCloud tool, a commercial tool for named entity recognition | MeaningCloud tool | Fee-based | None provided | None provided | None provided |
| Topaz 2016[64] | Lexicon-based | Lexicon-based approach using a range of diverse medical ontologies to map concepts to a hierarchical structure, which can be grouped to semantic types. | Treato | Fee-based | None provided | None provided | None provided |
| White 2014[66] | Lexicon-based | Queried terms corresponding to the drugs, conditions, and symptoms of interest were identified using sets of synonyms automatically generated from medical ontologies and historical search-result click data. | Not reported | Not reported | None provided | None provided | None provided |
| Whitman 2014[67] | Lexicon-based | Treato data were indexed using Unified Medical Language System, and a built-for-purpose "patient language” dictionary created by Treato researchers. Proprietary natural language processing (NLP) classification algorithms were used to index posts with this lexicon. | Treato | Open-access | None provided | None provided | None provided |
| Wu 2013[68] | Lexicon-based | Stemmed texts were matched to integrated knowledge-base | Integrated knowledge-base from SIDER, Drugs.com, DailyMed | Proprietary | None provided | None provided | None provided |
| Yang 2012a[69] | Lexicon-based | Based on the lexicon, events would be identified in threads by comparing a sliding window of tokens from the threads with each term in the lexicon from CHV. | Proprietary tool | Proprietary | None provided | None provided | None provided |
| Yang 2012b[70] | Lexicon-based | Concepts are detected and identified by matching a sliding window of tokens from threads with each item in the lexicon. | Not reported | Not reported | None provided | None provided | None provided |
| Yang 2013[71] | Lexicon-based | Lexicon-based approach using exact matching technique with a sliding window to match with drug names and adverse event lexicon, respectively. | Not reported | Not reported | None provided | None provided | None provided |
| Yang 2015b[73] | Lexicon-based | Lexicon-based approach to extract drug and adverse event entities using CHV. | Not reported | Not reported | None provided | None provided | None provided |
| Yates 2013b[75] | Dictionary | Used ADRTrace to mine terms and phrases that appeared in our MedSyn synonym set | ADRTrace | Proprietary | None provided | P: 69%, R: 89%, F: 78% | None provided |
| Yom-Tov 2013[76] | Lexicon-based | Lexicon generated from Wikipedia and ICD-10 symptoms. | Wikipedia, ICD-10 | Not applicable | None provided | None provided | None provided |
| ***Supervised classifier (n=6)*** | | | | | | | |
| Alvaro 2015[9] | Supervised classifier (NB, SVM, neural network, generalized linear model (GLM), and Bayesian GLM) | Used ML algorithms to classify tweets, but specific spans were not identified. | Caret package in R | Not applicable | None provided | None provided | None provided |
| Karimi 2015b[32] | Supervised classifier (CRF) | Used CRF classifier from the Stanford NER suite to identify spans of text that mention drugs, adverse reactions, diseases and symptoms to classify them based on their type. | Stanford NER suite | Open-access | A: 98% | A: 90% | None provided |
| Nikfarjam 2015[46] | Supervised classifier (CRF) | A supervised sequence labeling CRF classifier was used in ADRMine to extract the ADR concepts from user sentences. | CRF suite | Open access | None provided | None provided | None provided |
| Patki 2014[49] | Supervised classifier (NB, SVM), manual annotation | Comments were classified as adverse event or non-adverse event with Naive Bayes and SVM classifier, some documents were manually annotated for concepts. | Weka | Proprietary | None provided | None provided | Support Vector Machine A: 82.6%, F: 54.0% Naïve Bayesian A: 77.6%, F: 65.2% |
| Sarker 2016a[56] | Supervised classifier (CRF), word embeddings | DLIR system based on Conditional Random Fields (CRFs) trained on the annotated data. | word2vec | Open-access | None provided | None provided | CRF-based DLIR system P: 76.0%, R: 51.1%, F: 61.1% NTTUMUNSW extraction system P: 78.2%, R: 41.2%, F: 54.0% |
| Yates 2013a[74] | Supervised classifier (CRF) | Used dependency relationships to determine which path can be followed to generate candidate adverse event. Each sequence of shortest paths in a potential adverse event is then turned into binary features for use with a Conditional Random Field (CRF). | Stanford Parser, CRF suite | Open-source (Stanford Parser), Open-access (CRF suite) | None provided | None provided | ADRTrace P: 39%, R: 61%, F: 47.6% ADRTrace+DepADR P: 39%, R: 65%, F: 48.8% DepADR P: 61% R: 32%, F: 42% Sliding window P: 32% R: 74%, F: 44.7% |
| ***Lexicon-based and supervised classifier (n=2)*** | | | | | | | |
| Jimeno-Yepes 2015[29] | Lexicon-based, Supervised classifier (CRF) | Three methods were used to annotate the data set with the three entity types: MetaMap with and without word sense disambiguation (WSD); custom NER tagger Micromed with CRFSuite; and Stanford NER tagger (SNER) with CRFSuite. | MetaMap, Micromed, Standard NER tagger (SNER) | Open-access (MetaMap and SNER), Proprietary (Micromed) | Exact match P: 82.1%, R: 41.2%, F: 54.9% Partial match P: 72.2%, R: 60.4%, F: 65.8% | Exact match P: 72.2%, R: 60.4%, F: 65.8% Partial match P: 74.5%, R: 62.3%, F: 67.9% | None provided |
| Metke-Jimenez 2015[43] | Lexicon-based, Supervised classifier (CRF) | A variety of dictionary-based approaches (MetaMap [baseline], VSM + UMLS, VSM + CHV, VSM + SCT, SNOMED CT, VSM + AMT) | Lucene search engine, Stanford NER suite | Open-access | VSM+UMLS P: 16.0%, R: 88.2%, F: 27.1%, A: 54.6% VSM+AMT P: 16.0%, R: 77.5%, F: 26.6%, A: 58.9%  MetaMap P: 2.2%, R: 2.1%, F: 2.1%, A: 81.6%  VSM+CHV P: 46.8%, R: 85.6%, F: 60.5%, A: 89.3%  CRF P: 94.3%, R: 84.0%, F: 88.9%, A: 98.0% | VSM+UMLS P: 26.4%, R: 39.2%, F: 31.6%, A: 45.4% MetaMap P: 10.5%, R: 8.0%, F: 9.1%, A: 48.5% VSM+CHV P: 45.7%, R: 37.0%, F: 40.9%, A: 65.6%  VSM+SCT P: 49.8%, R: 35.2%, F: 41.2%, A: 67.8% CRF P: 64.4%, R: 56.5%, F: 60.2%, A: 76.0% | None provided |
| ***Rule-based phrase extraction (n=2)*** | | | | | | | |
| Liu 2011[35, 78] | Phrase extraction | Phrases automatically extracted from data and filtered, then manually grouped. | Not reported | Not applicable | SVM baseline A: 84.4%, P: 82.9%, R: 97.8%, F: 89.7% SVM + Log likelihood ratio terms A: 87.1%, P: 86.3%, R: 96.8%, F: 91.2% SVM baseline wihout drug names A: 78.4%, P: 76.8%, R: 98.6%, F: 86.3% SVM + Log likelihood ratio terms without drug names A: 80.1%, P: 80.6%, R: 95.6%, F: 87.4% | None provided | None provided |
| Risson 2016[52] | Rule-based | Rule-based phrase identification based on a set of predefined categories. | Not reported | Not reported | None provided | None provided | None provided |
| ***Sentiment analysis(n=2)*** | | | | | | | |
| Akay 2015[8] | Term frequency, sentiment analysis | Text data containing the highest TF-IDF scores were tagged with a modified NLTK toolkit using MATLAB to ensure that they reflected the negativity of a negative word and the positivity of a positive word in context. | NLTK toolkit using MATLAB, Proprietary program | Open-access (NLTK toolkit); Fee-based (MATLAB); Proprietary tool | None provided | None provided | None provided |
| Chee 2009[16] | Sentiment analysis | Messages were matched against the Linguistic Inquiry and Word Count (LIWC) lexicon categories and emotions. | Linguistic Inquiry and Word Count (LIWC) | Fee for service | None provided | None provided | None provided |
| ***Statistical model (n=2)*** | | | | | | | |
| Metke-Jimenez 2015[43] | CRF, dictionary | Hybrid methods using conditional random fields to identify relevant text spans prior to use of a dictionary-based method (CRF + VSM and CRF + Ontoserver) | Lucene search engine, Stanford NER suite | Open-access | CRF P: 94.3%, R: 84.0%, F: 88.9%, A: 98.0% | CRF P: 64.4%, R: 56.5%, F: 60.2%, A: 76.0% | None provided |
| Yang 2015a[72] | Topic Model (LDA) | LDA topic modeling to identify the topic of each post, k-means clustering to clean up positive and negative examples. | Not reported | Not applicable | None provided | None provided | None provided |
| ***Not specified (n=1)*** | | | | | | | |
| Rizo 2011[53] | Unspecified | OpenCalais to map medical entities | OpenCalais | Fee-based | None provided | None provided | None provided |

**Abbreviations:** P - Precision; R - Recall; F - F-measure; A - Accuracy

# Appendix 13. Drug name normalization methods and results

| **Article ID** | **Drug name normalization approach** | | **Method description** | **Tool or software used** | **Tool accessibility** | **Validity & reliability results** |
| --- | --- | --- | --- | --- | --- | --- |
| ***Dictionary or lexicon-based approaches (n=19)*** | | | | | | |
| Benton 2011[10] | | Lexicon-based | Medical vocabulary for dietary supplements were author-compiled, and for pharmaceuticals the Cerner Multum’s Drug Lexicon was consulted and augmented with Consumer Health Vocabulary (CHV), four drugs manually selected for evaluation | Drug dictionary compiled using Cerner Multum’s Drug Lexicon, CHV and web | Proprietary dictionary | None provided |
| Correia 2016[19] | | Dictionary | Drug dictionary from a previously developed pharmacokinetics ontology using only FDA-approved drugs, along with generic names and their synonyms (found in DrugBank). The natural product dictionary was built from MedlinePlus terms for herbal medicines. A Cannabis dictionary was assembled by web searching for known synonyms of cannabis. | Drug dictionary based on FDA, DrugBank, MedlinePlus and web search | Proprietary dictionary | None provided |
| Elhadad 2014[20] | | Lexicon-based | RxNorm used as a seed for lexicon expansion. Candidates identified from out-of-vocabulary words according to Aspell, mapped to medication names with context vector similarity. | Drug dictionary based on RxNorm, Aspell | Proprietary dictionary available at www.people.dbmi.columbia.edu/noemie | None provided |
| Freifeld 2014b[22] | | Dictionary | A researcher compiled product dictionary which includes both brand names and generic names as separate product entries in the taxonomy. | Proprietary dictionary | Proprietary dictionary | None provided |
| Ginn 2014 [23, 81] | | Dictionary with phonetic matching | Dictionary-based method in which the dictionary was supplemented by a phonetically derived list of possible misspellings of drug names. | Not reported | Not reported | None provided |
| Gupta 2014[24] | | Dictionary, fuzzy matching | Drugs and treatments (DT) dictionary constructed from Wikipedia's list of drugs, surgeries and delivery devices, RxList, MedLinePlus, Medicinenet, MeDRA, NCI Thesaurus. | Drug dictionary based on Wikipedia, RxList, MedLinePlus, Medicinenet, MeDRA, NCI Thesaurus | Proprietary dictionary | None provided |
| Hadzi-Puric 2012[25] | | Dictionary | Mapped drug names to appropriate Anatomical Therapeutic Chemical (ATC) code | Anatomical Therapeutic Chemical (ATC) | Open-access | None provided |
| Karimi 2015b[32] | | Dictionary | Ontoserver used to map drugs to AMT (Australian Medicines Terminology) ontologies. | Ontoserver with SNOMED CT and AMT | Open-access version is Shrimp | A: 92% |
| Liu 2015a[37, 79, 80] | | Lexicon-based | MetaMap was used to identify medical concepts in UMLS from health social media. Configured MetaMap to recognize the terms that belong to the Chemicals and Drugs semantic group for drug name entities. Filtered results from MetaMap with drug names and event names in FDA’s drug safety database FAERS. | UMLS-MetaMap, FAERS | Open-access | None provided |
| Liu 2015b[38] | | Lexicon-based | MetaMap was used to identify medical concepts in UMLS from health social media. Configured MetaMap to recognize the terms that belong to the Chemicals and Drugs semantic group for drug name entities. Filtered results from MetaMap with drug names and event names in FDA’s drug safety database FAERS. | UMLS-MetaMap, FAERS | Open-access | None provided |
| Metke-Jimenez 2015[43] | | Lexicon-based and dictionary | A variety of dictionary-based approaches (MetaMap [baseline], VSM + UMLS, VSM + CHV, VSM + SCT, SNOMED CT, VSM + AMT). | Lucene search engine, Stanford NER suite | Open-access | (MetaMap) A: 0% (VSM+UMLS) A: 0% (VSM+CHV) A: 0% (CRF+VSM) A: 74.9% (CRF+Ontoserver) A: 77.3% (VSM+AMT) A: 75.8% |
| Powell 2016[51] | | Dictionary | Automated classification process to interpret the wording used to describe drug and medical condition concepts using natural language processing (NLP) software | Anatomical Therapeutic Chemical (ATC) classification system, NLP software | Open-access (dictionary), Proprietary (NLP system) | None provided |
| Sampathkumar 2014[54] | | Lexicon-based | Drug dictionary compiled from drugs.com to match to lexical ID | Drug dictionary compiled from drugs.com | Proprietary dictionary | None provided |
| Sarker 2015[55] | | Lexicon-based, phonetic matching | Used UMLS-MetaMap to identify concept IDS (CUIs). The CUIs provide more fine grained categorization of medical concepts so that different lexical representations of the same concept can be identified. | UMLS-MetaMap | Open-access | None provided |
| Sarker 2016b[57] | | Dictionary, phonetic matching | A dictionary compiled a set of colloquial phrases and terms that are used to discuss drugs from NoSlang.com | Drug-slang dictionary compiled from NoSlang.com | Proprietary dictionary | None provided |
| Segura-Bedmar 2014[61, 82] | | Lexicon-based | A researcher developed drug dictionary compiled from CIMA, ATC system and Vademecum | Proprietary dictionary using CIMA, ATC system and Vademecum; MeaningCloud with GATE (gazetteers) plug-in | Fee-based | None provided |
| White 2014[66] | | Lexicon-based | Queried terms corresponding to the drugs, conditions, and symptoms of interest were identified using sets of synonyms automatically generated from medical ontologies and historical search-result click data. | Not reported | Not reported | None provided |
| Wu 2013[68] | | Lexicon-based | Matched stemmed texts to integrated knowledgebase derived from SIDER, Drugs.com, DailyMed | Drug dictionary derived from SIDER, Drugs.com, DailyMed | Proprietary dictionary | None provided |
| Yang 2015b[73] | | Lexicon-based | List of drugs lexicon built from CHV | CHV | Open-access | None provided |
| ***Statistical models (n=1)*** | | | | | | |
| Metke-Jimenez 2015[43] | CRF, dictionary | | Hybrid methods using conditional random fields to identify relevant text spans prior to use of a dictionary-based method (CRF + VSM and CRF + Ontoserver). | Lucene search engine, Stanford NER suite | Open-access | (CRF+VSM) A: 74.9% (CRF+Ontoserver) A: 77.3% |
| **Abbreviations:** A - Accuracy | | | | | | |

# Appendix 14. Medical event normalization methods and results

| **Article ID** | **Medical entity normalization approach** | | **Method description** | | **Tool or software used** | **Tool accessibility** | **Validity & reliability results** | |
| --- | --- | --- | --- | --- | --- | --- | --- | --- |
| ***Dictionary or lexicon-based approaches (n=33)*** | | | | | | | | |
| Akay 2015[8] | Dictionary | | | Used the National Library of Medicine’s Medical Subject Heading (MeSH), a controlled vocabulary and a custom designed program to map words in the forum to the MeSH database. Some synonyms mapped manually from dictionaries. | National Library of Medicine’s Medical Subject Heading (MeSH); proprietary program | Open-access (dictionary), Proprietary program | None provided | |
| Benton 2011[10] | Lexicon-based, stemming | | | Terms stemmed with the Porter stemmer and then matched to the lexicon. Medical vocabulary for events were generated using MedicineNet, FAERS database and Consumer Health Vocabulary | Natural Language Toolkit (NLTK) Porter Stemmer | Proprietary | None provided | |
| Bian 2012[12] | Dictionary | | | Used Metamap to map concepts to Unified Medical Language Systems (UMLS) developed at the National Library of Medicine (NLM) | MetaMap | Open-access | None provided | |
| Carbonell 2015[14] | Dictionary, fuzzy matching | | | Used the BeFree System's Biomedical Named Entity Recognition (BioNER) module for diseases dictionary collected from the Unified Medical Language System (UMLS) | BeFree System BioNER module | Unclear | None provided | |
| Chary 2014[15] | Dictionary | | | The distance between clusters in WordNet was used to quantify similarities between two concepts. | WordNet | Open-access | None provided | |
| Chee 2011[17] | Lexicon-based | | | Dictionary derived from MedDRA, and lists of diseases. | Dictionary derived from MedDRA and list of diseases | Proprietary | None provided | |
| Elhadad 2014[20] | Lexicon-based | | | UMLS and SIDER used as a seed for lexicon expansion. Terms mapped to semantic categories using context vector similarity clustering method. | Dictionary derived from UMLS and SIDER | Proprietary dictionary available at www.people.dbmi.columbia.edu/noemie | None provided | |
| Freifeld 2014a[21] | Dictionary | | | A dictionary was developed to translate Internet vernacular to MedDRA terms. | MedDRA | Open-access to regulatory agencies and non-commercial organizations | None provided | |
| Freifeld 2014b[22] | Dictionary | | | Researcher compiled symptom dictionary which contained colloquial symptom categories linked to respective MedDRA terms. | Proprietary dictionary | Proprietary | None provided | |
| Gupta 2014[24] | Dictionary, fuzzy matching | | | Symptoms and conditions dictionary automatically constructed from: MedLinePlus, MedicineNet, MeDRA, CHV. | Dictionary derived from MedLinePlus, MedicineNet, MeDRA, CHV | Proprietary | None provided | |
| Hadzi-Puric 2012[25] | Dictionary | | | Dictionary compiled based on three resources: European Agency for the Evaluation of Medical Products (EMEA), Medicines and Medical Devices Agency of Serbia (ALIMS), Drug Bank. MetaMap was used to map concepts in biomedical text to concepts in the UMLS Metathesaurus. | Dictionary derived from European Agency for the Evaluation of Medical Products (EMEA), Medicines and Medical Devices Agency of Serbia (ALIMS), Drug Bank; MetaMap | Proprietary dictionary; Open-access(MetaMap) | None provided | |
| Karimi 2015b[32] | Dictionary | | | Ontoserver used to map extracted symptoms to SNOMED CT ontologies. Mapping between the concepts and MedDRA was done through SNOMED CT. Used an in-house mapping table between SNOMED CT concepts to MedDRA to find equivalents of SNOMED CT concepts in MedDRA. | Ontoserver with SNOMED CT and AMT, MedDRA | Open-access version is Shrimp | A: 67% | |
| Leaman 2010[34] | Lexicon-based, stemming, fuzzy matching | | | A researcher developed dictionary compiled from COSTART subset (UMLS Metathesaurus), SIDER, Canada Drug Adverse Reaction Database, colloquial phrases from DailyStrength. Concepts were matched to lexicon using a sliding window. | Dictionary derived from COSTART subset (UMLS Metathesaurus), SIDER, Canada Drug Adverse Reaction Database, colloquial phrases from DailyStrength | Proprietary dictionary | None provided | |
| Liu 2014[36] | Lexicon-based | | | Expanded medical lexicon with colloquial terms from the Consumer Health Vocabulary corresponding to standard medical terms using MetaMap. | Consumer Health Vocabulary; MetaMap | Open-access | None provided | |
| Liu 2015a[37, 79, 80] | Lexicon-based | | | MetaMap was used to identify medical concepts in UMLS from health social media. Configured MetaMap to match the terms that belong to the Disorders group for standard adverse event entities. Also used CHV to recognize consumer-preferred terms which is normalized to standard medical terms. | UMLS-MetaMap, CHV | Open-access | None provided | |
| Liu 2015b[38] | Lexicon-based | | | MetaMap was used to identify medical concepts in UMLS from health social media. Configured MetaMap to match the terms that belong to the Disorders group for standard adverse event entities. Also used CHV to recognize consumer-preferred terms which is normalized to standard medical terms. | UMLS-MetaMap, CHV | Open-access | None provided | |
| Metke-Jimenez 2014[42] | Lexicon-based, stemming | | | Used controlled vocabularies to identify text fragments that refer to relevant concepts. The following controlled vocabularies were used: CHV, UMLS. | CHV, UMLS | Open-access | None provided | |
| Metke-Jimenez 2015[43] | Lexicon-based and dictionary | | | A variety of dictionary-based approaches were used: MetaMap (baseline), VSM + UMLS, VSM + CHV, VSM + SCT, SNOMED CT, VSM + AMT | Lucene search engine, Stanford NER suite | Open-access | (MetaMap) A: 2.9% (VSM+UMLS) A: 10.5% (VSM+CHV) A: 10.6% (CRF+VSM) A: 32.7% (VSM+SCT) A: 33.2% (CRF+Ontoserver) A: 37.6% | |
| Nikfarjam 2015[46] | Lexicon-based, lemmatization | | | Compiled an exhaustive list of ADR concepts and their corresponding UMLS IDs based on COSTART, SIDER, subset of CHV which was mapped using Lucene. | Dictionary based on COSTART, SIDER, subset of CHV; CRFsuite, Dragon toolkit and WordNet for lemmatization, Apache Lucene | Proprietary dictionary available at http://diego.asu.edu/downloads/publications/ADRMine/ADR_lexicon.tsv | None provided | |
| Powell 2016[51] | Dictionary | | | Automatic process not described. MedDRA dictionaries used in conjunction with partial manual assistance to map products to symptoms. | Medical Dictionary for Regulatory Activities (MedDRA), NLP software | Open-access to regulatory agencies and non-commercial organizations (MedDRA), Proprietary (NLP software) | None provided | |
| Sampathkumar 2014[54] | Lexicon-based | | | Side-effects dictionary compiled from SIDER to map to lexicon ID | Side effects dictionary made from SIDER | Proprietary | None provided | |
| Sarker 2015[55] | Lexicon-based, stemming | | | Used UMLS-MetaMap to identify concept IDS (CUIs). Also topic modeling with Mallet. | UMLS-MetaMap, Mallet | Open-access | None provided | |
| Topaz 2016[64] | Dictionary | | | Used a diverse range of medical ontologies (including MedDRA), organized in a concept-based structure and codes similar to the Unified Medical Language System (UMLS) and patient language vocabularies for the concept extraction and analysis. | Treato | Fee-based | None provided | |
| White 2014[66] | Lexicon-based | | | Queried terms corresponding to the drugs, conditions, and symptoms of interest were identified using sets of synonyms automatically generated from medical ontologies and historical search-result click data. | Not reported | Not reported | None provided | |
| Whitman 2014[67] | Dictionary | | | Treato data were indexed using Unified Medical Language System, and a built-for-purpose "patient language" dictionary created by Treato researchers. Proprietary natural language processing (NLP) classification algorithms were used to index posts with this lexicon, resulting in a dataset that may be easily searched and analyzed in aggregate. | Treato using UMLS | Fee-based | None provided | |
| Wu 2013[68] | Lexicon-based, stemming | | | Matched stemmed texts to integrate knowledge-based derived from SIDER. | Dictionary derived from SIDER | Proprietary | None provided | |
| Yang 2012a[69] | Dictionary | | | Lexicon generated based on Consumer Health Vocabulary (CHV) and FAERS to match with the dataset. | Dictionary derived from FAERS and CHV | Proprietary | None provided | |
| Yang 2012b[70] | Dictionary | | | Used Consumer Health Vocabulary (CHV) to generate ADR lexicon. | CHV | Open-access | None provided | |
| Yang 2013[71] | Dictionary | | | Used Consumer Health Vocabulary (CHV) to generate ADR lexicon. | CHV | Open-access | None provided | |
| Yang 2015b[73] | Dictionary | | | ADRs lexicon built from Consumer Health Vocabulary (CHV). | CHV | Open-access | None provided | |
| Yates 2013a[74] | Lexicon-based | | | MedSyn was derived from a subset of the Unified Medical Language System Metathesaurus (UMLS). MedSyn was used to treat different terms or phrases that expressed the same ADR as equivalent. | MedSyn derived from UMLS | Proprietary | None provided | |
| Yates 2013b[75] | Dictionary | | | Researcher developed synonym set (MedSyn) based on UMLS and SIDER 2. | Proprietary dictionary MedSyn | Proprietary | None provided | |
| Yom-Tov 2013[76] | Lexicon-based, synonyms | | | Synonyms were automatically extracted from similar web searches and annotated by professionals; the full set of expansion terms was added to the lexicon. | Dictionary derived from ICD-10 and Wikipedia | Proprietary | None provided | |
| ***Statistical model (n=2)*** | | | | | | | | |
| Metke-Jimenez 2015[43] | | CRF, dictionary | | Hybrid methods using conditional random fields to identify relevant text spans prior to use of a dictionary-based method (CRF + VSM and CRF + Ontoserver) | Lucene search engine, Stanford NER suite | Open-access | | (CRF+VSM) A: 32.7% (CRF+Ontoserver) A: 37.6% |
| Yang 2015a[72] | Topic Model (LDA) | | | Symptoms and ADRs identified by Latent Dirichlet Allocation (LDA) and refined by k-means clustering | Not reported | Not reported | | None provided |
| **Abbreviations:** A - Accuracy | | | | | | | | |

# Appendix 15. Relation extraction methods and results

| **Article ID** | **Relation extraction approach** | **Method description** | **Tool or software used** | **Tool accessibility** | **Validity & reliability results** |
| --- | --- | --- | --- | --- | --- |
| ***Rule-based or statistical association mining (n=16)*** | | | | | |
| Benton 2011[10] | Association mining | Association mining based on co-occurrence of terms. | Not reported | Not reported | All drugs P: 35.1%, R: 77%, F: 48.2% Nolvadex P: 42.4%, R: 79.1%, F-measure: 55.3% Arimidex P: 36.8%, R: 75.0%, F: 49.4% Aromasin P: 25.0%, R: 5.8%, F: 9.4% Femara P: 35.6%, R: 78.0%, F: 48.9% |
| Carbonell 2015[14] | Statistical association | Analyzed co-evolution of drug mentions on Twitter to test if those pairs of drugs that were often found simultaneously mentioned could correspond to cases of known drug-drug interactions using time-series analysis. Also analyzed the co-occurrence of disease condition with drug names in the messages, as well as the corresponding drug categories. | R software | Open-access | None provided |
| Correia 2016[19] | Statistical association | A symmetric co-occurrence graph is computed for time-window resolutions w = 1 month, 1 week and 1 day to assess correlation of entities mentioned together. | Not reported | Not reported | None provided |
| Hadzi-Puric 2012[25] | Statistical association | Used several disproportionality analysis methods to quantify drug and adverse reaction association: reporting ratio (RR), proportional reporting ratios (PRR), reporting odds ratios (ROR) and information component (IC). | Not reported | Not reported | None provided |
| Karimi 2015b[32] | Association mining | Employed association rule mining based on support and confidence of a potential rule (relationship between two extracted concepts). | Not reported | Not reported | None provided |
| Liu 2011[35] | Statistical association | Used log-likelihood ratio to measure association between drug class and side-effects. | Not reported | Not reported | None provided |
| Nikfarjam 2011[45] | Association mining | Association rule mining using Apriori algorithm. | Apriori tool | Open-access | P: 70.01%, R: 66.32%, F: 67.96% |
| Rizo 2011[53] | Statistical association | The OpenCalais™ procedure was used to calculate reliability scores that indicate the extent of a reliable association between a reported symptom and a drug. | OpenCalais | Fee-based | None provided |
| Topaz 2016[64] | Statistical association | Generated frequency-based rankings for each distinct reaction and used Pearson correlation metrics in STATA to analyze the potential association between the frequency of adverse event reports in clinical and social media data. | STATA | Fee-based | None provided |
| White 2013[65] | Statistical association | Used conditional disproportionality analysis to assess the increased chance of a user searching for hyperglycemia-related terms given that they searched for both pravastatin and paroxetine. Reporting ratios (RR) were computed based on observed versus expected adverse reports. | Not reported | Not reported | (All terms) AUC: 0.82 (Focused terms) AUC: 0.74 |
| White 2014[66] | Statistical association | Association statistic called the query rate ratio (QRR) between drugs and outcomes of interest was used. | Not reported | Not reported | *Association statistic (search logs)* AUC: 0.73 for acute myocardial infarction AUC: 0.92 for upper gastrointestinal bleeding AUC: 0.81 for acute liver grievance AUC: 0.88 for acute renal failure Average AUC: 0.83 *Association statistic (search logs + FAERS)* AUC: 0.75 for acute myocardial infarction AUC: 0.92 for upper gastrointestinal bleeding AUC: 0.86 for acute liver grievance AUC: 0.93 for acute renal failure Average AUC: 0.86 |
| Yang 2012a[69] | Association mining | Association rule mining using sliding window to measure support, confidence and leverage of the association was performed. | Not reported | Not reported | None provided |
| Yang 2012b[70] | Association mining | Used association mining to calculate leverage and lift for each possible pair of drugs and adverse reactions in the dataset, and calculated proportional reporting ratio. | Not reported | Not reported | R: 71.4% R: 66.6% R: 71.4% |
| Yang 2013[71] | Association mining | Used association mining technique to detect drug-to-drug interactions based on the co-occurrence of the pair of drugs and their consequential adverse reaction in the posts or comments using measures of support, confidence, leverage, and lift. | Not reported | Not reported | P: 45%, R: 80%, F: 57.6% P: 55%, R: 93%, F: 69.1% P: 60%, R: 100%, F: 75% |
| Yang 2015b[73] | Association mining, tensor decomposition | Two methodologies: matrix-based technique based on association rule-mining with temporal analysis to identify correlations between drugs and adverse events, and tensor decomposition technique to capture hidden signal strength of drug-adverse event associations using three-dimensional model (drug x ADR x time at the same time) without having to collapse the dataset into matrices. | Not reported | Not reported | 1-year time window without overlapping (Matrix-based) R: 50% (Tensor-based) R: 61% 2-year time window with 1-year overlap (Matrix-based) R: 54% (Tensor-based w/ complete data) R: 50% (Tensor-based w/ incomplete data) R: 61% |
| Yom-Tov 2013[76] | Statistical association | For each drug-symptom pair, a 2-way contingency table was constructed to count the number of times a symptom was searched by users who did and did not search for the drug. Prevalence of each symptom was scored as a reaction to the drug using the Pearson’s goodness of fit test, the chi-square test statistic; this score as the query log reaction score (QLRS). | Not reported | Not reported | F: 44%-94%, AUC: 0.57-0.71 |
| ***Supervised classifier (n=14)*** | | | | | |
| Bian 2012[12] | Supervised classifier (SVM) | Two-class Support Vector Machine (SVM) to identify side effects caused by the use of the drugs based on the positive cases identified in the previous model. | SVM | Proprietary | A: 74%, AUC: 0.74 |
| Chee 2011[17] | Supervised classifier (NB, SVM) | Naïve Bayes (NB) and Support Vector Machine (SVM) classifier with a RBF kernel were the two base classification algorithms used. | Not reported | Not reported | None provided |
| Freifeld 2014b[22] | Supervised classifier (NB, Fisher-Robinson) | Employed a statistical approach for automated document classification using Fisher-Robinson classifier (adapted the HealthMap statistical document classification algorithm) and Naïve Bayes Classifier. | Not reported | Not reported | Twitter P: 68%, R: 89%, F: 77% Facebook P: 46%, R: 69%,F: 55% |
| Ginn 2014[23, 81] | Supervised classifier (NB, SVM) | Used two supervised machine learning algorithms for the binary classification task of identifying tweets hat contain adverse event mentions and those that don't. Used Naive Bayes and Support Vector Machine learning approach. | Not reported | Open-access | Naïve Bayesian (balanced dataset) A: 74.6% (40/60) A: 74.6% (30/70) A: 75.2% Support Vector Machine (balanced dataset) A: 71.5% (40/60) A: 72.8% (30/70) A: 76.6% |
| Liu 2014[36] | Semi-supervised classifier (SVM) | Two subtasks: relation detection (semi-supervised machine learning) and relation classification (rule-based algorithm). | Stanford Parser , Transductive SVM-light, NegEx | Open-source | A: 29.44%, P: 29.44%, R: 100%, F: 45.49% A: 63.20%, P: 42.74%, R: 73.53%, F: 54.06% A: 80.74%, P: 65.36%, R: 73.53%, F: 69.20% |
| Liu 2015a[37, 79, 80] | Semi-supervised classifier (SVM) | Developed a shortest dependency path kernel based statistical learning method to determine relationship between drug and a medical event. Utilized Support Vector Machines (SVM) to learn patterns from posts with related drugs and events. Also applied semantic filtering to detect negations in medical documents. | Stanford Parser, Stanford CoreNLP packages, SVM-light, NegEx | Open-access | American Diabetes Association P: 82.0%, R:56.5%, F: 66.9% Diabetes Forums P: 78.6%, R:60.4%, F: 62.2% Diabetes Forum P: 75.2%, R:58.0%, F: 65.5% MedHelp (heart disease) P: 80.7%, R:65.3%, F: 72.2% |
| Liu 2015b[38] | Distant supervised learning (MIML) | Relation extraction was conducted at the sentence level. Co-reference resolution was incorporated to link medical entities across sentences within the same document to identify potentially related medical events and medications mentioned across sentences. Distant supervised learning was then used for binary relation extraction between medications and medical conditions based on the multi-instance multi-label assumption. | Not reported | Not reported | P: 70% |
| Nikfarjam 2015[46] | Supervised classifier (CRF) | A supervised sequence labeling CRF classifier was used in ADRMine to extract the ADR concepts from user sentences based on semantic cluster features from word embeddings. | CRFsuite, word2vec (for word embeddings) | Open-access | ADRMine (without cluster classifier): DailyStrength P: 87.4%, R: 72.3%, F: 79.1%  ADRMine (with cluster classifier): DailyStrength P: 86.0%, R: 78.4%, F: 82.1%  ADRMine (without cluster classifier): Twitter P: 78.8%, R: 54.9%, F: 64.7% ADRMine (with cluster classifier): Twitter P: 76.5%, R: 68.2%, F: 72.1% |
| Powell 2016[51] | Supervised classifier (NB), Manual | Bayesian probabilistic model was developed through statistical machine learning computation, based on Robinson’s approach to assign each post an indicator score on a scale of 0–1 by the algorithm (0 = low probability that post was an AE, 1 = high probability that the post was an AE), followed by manual annotation. | Not reported | Not reported | P: 50%; R: 92% |
| Sampathkumar 2014[54] | Supervised classifier (HMM) | Used the Hidden Markov Model (HMM) classifier to predict the presence of relationship between a drug and an adverse event. | Not reported | Not reported | (All components) P: 78%, R: 74.5%, F: 76% (No HTML filter) P: 78%, R: 74%, F: 75.7% (No plain text filter) P: 63%, R: 27.1%. F: 37.8% |
| Sarker 2015[55] | Supervised classifier (NB, SVM, ME) | Binary classification of text segments into adverse event or non-adverse event categories was performed using three supervised classification approaches: Naïve Bayes (NB), Support Vector Machines (SVM) and Maximum Entropy (ME). | Weka, LibSVM | Open-access | (ADE) F: 81.2% (Twitter) F: 53.8% (DailyStrength) F: 67.8% |
| Sarker 2016a[56] | Supervised classifier (NB, SVM, Random Forest) | Systems using supervised classification approaches:  Mayo-NLP system with a feature set containing unigrams, bigrams, and trigrams (a selected list, using mutual information), co-occurrence of drug and side effect, negation, and sentiment score were used to train Random Forest classifiers for identifying ADR  TJZZF system with (1) a concept-matching classifier based on an ADR lexicon, (2) a maximum entropy (ME) classifier with n-gram features and a TF.IDF weighting scheme, (3) a ME classifier based on n-grams using Naive Bayes (NB) log-count ratios as feature values, and (4) a ME classifier with word embedding features. | Not reported | Not reported | P: 35.5%, R: 30.2%, F: 32.7%, A: 90.4% P: 35.3%, R: 51.2%, F: 41.8%, A: 89.0% P: 34.0%, R: 37.9%, F: 35.8%, A: 89.5% P: 36.1%, R: 50.1%, F: 41.9%, A: 89.3% P: 20.2%, R: 74.3%, F: 31.7%, A: 75.4% |
| Sarker 2016b[57] | Supervised classifier (NB, SVM, ME, Decision Tree) | Used four off-the-shelf supervised classification algorithms to assess the performance of automatic detection, namely: Naive Bayes, Support Vector Machines (SVMs), Maximum Entropy (ME), and a decision tree-based classifier. | LibSVM, Weka | Open-access | P: 41%, R: 51%, F: 46%, A: 82% |
| Wu 2013[68] | Supervised classifier (Rocchio), Statistical Association | Discriminative Classification Method using Rocchio method and Generative Modeling method was used. | Not reported | Not reported | P: 69%, R: 23% P: 70%, R: 69% |
| Yang 2015a[72] | Supervised classifier (SVM) | Latent Dirichlet Allocation modeling module and a partially supervised classification approach were employed; records were subsequently classified with an SVM. | Not reported | Not reported | Biaxin F: 70.7% Lansoprazole F: 73.3% |
| Yates 2013a[74] | Supervised classifier (CRF), Dependency parses | Relationship between drug and an event was detected using CRF. | CRFsuite | Open-access | P: 63%, R: 36% |
| ***Dictionary or lexicon-based approach (n=4)*** | | | | | |
| Chary 2014[15] | Lexicon-based | Word frequency by plateau and semantic similarity were calculated to find keywords semantically related to the medication. | Not reported | Not reported | None provided |
| Segura-Bedmar 2014[61, 82] | Lexicon-based vs. association mining | Lexicon-based approach using the SpanishDrug-EffectBD database. Relations were then extracted using Textalytics and GATE gazetteers. | MeaningCloud with GATE (gazetteers) plug-in | Fee-based | SpanishDrugEffectBD based relation extraction (Overall) P: 83%, R: 15%, F: 25% (Rx indication) P: 50%, R: 2%, F: 3% (ADR) P: 65%. R: 11%, F: 18% Co-occurrence approach (Overall) P: 44%, R: 84%, F: 58% |
| Segura-Bedmar 2015[62] | Lexical context | Used the Shallow Linguistic (SL) kernel, Global Context and Local Context for relation extraction. | Proprietary system | Proprietary | P: 48%, R: 59%, F: 53% |
| Freifeld 2014a[21] | Dictionary mapping | NLP semi-automated classifier – system used. | Not reported | Not reported | None provided |
| ***Sentiment analysis (n=2)*** | | | | | |
| Akay 2015[8] | Sentiment analysis (network analysis) | Consumer sentiment analysis performed using self-organizing maps (SOM) to analyze word frequency data derived from users’ forum posts. | SOM toolbox for MATLAB | MATLAB is fee-based | None provided |
| Chee 2009[16] | Sentiment analysis | Counts containing number of positive emotion words, and negative ones, and total number of words were recorded. The negative ratio of negative emotion words to total words in a message was used to determine negative valence. | Not reported | Not reported | None provided |

**Abbreviations:** P - Precision; R - Recall; F - F-measure; A - Accuracy; AUC - Area under the curve

# Appendix 16. Additional processing methods and results

| **Article ID** | **Additional steps** | **Validity & reliability results** |
| --- | --- | --- |
| ***Processing non-English social media posts (n=5)*** | | |
| Chary 2014^[15]^ | **Non-English text processing:** Non-English texts were included to the extent that it appeared in ASCII characters. | None provided |
| Hadzi-Puric 2012[25] | **Non-English text processing:** POS tagger for Serbian TnT. | None provided |
| Metke-Jimenez 2015[43] | **Non-English text processing:** Stanford NER used for Arabic, Chinese, French, German, and Spanish languages. | None provided |
| Segura-Bedmar 2014[61, 82] | **Non-English text processing:** MedDRA is a multilingual medical terminology dictionary supporting the following languages: Chinese, Czech, Dutch, French, German, Hungarian, Italian, Japanese, Portuguese, and Spanish. SpanishDrug-EffectBD was automatically built with information about drugs, their drug indications as well as their adverse drug reactions in Spanish. | None provided |
| Segura-Bedmar 2015[62] | **Non-English text processing:** MeaningCloud is a multilingual text analysis engine | None provided |
| ***Identifying first hand experiences from witnesses (n=4)*** | | |
| Alvaro 2015[9] | **Report Source Classification:** to distinguish first-hand experiences, second-hand experiences and other information, author developed a feature-based classifier using C50, SVM using a linear kernel (SVM), Naive Bayes (NB), Multi-Layer Perceptron (MLP), Generalized Linear Model (GLM), and Bayesian Generalized Linear Model (BGLM) from R’s Caret package. | F: 64%, Informedness: 43% |
| Bian 2012[12] | **Report source classification:** implemented a Support Vector Machine (SVM) to label a collection of Tweets to determine if it is a firsthand or second-hand experience. | A: 74%, AUC: 0.82 |
| Liu 2014[36] | **Report Source Classification:** developed a feature-based classifier using Transductive SVM-light with linear kernel for semi-supervised learning-based classification. | (without RSC) P: 52.73%, R: 100%, F: 69.05% (with RSC) P: 89.65%, R: 91.42%, F: 90.53% |
| Liu 2015a^[37, 79, 80]^ | **Report source classification:** developed a feature-based classification model to distinguish patient reports from hearsay. Adopted bag of words (BOW) features and Transductive Support Vector Machines for classification. | American Diabetes Association (with RSC) P: 83.9%, R:84.3%, F:84.1% (without RSC) P: 59.7%, R:100%, F: 74.8% Diabetes Forums (with RSC) P: 87.2%, R:83.1%, F:85.1% (without RSC) P: 56.0%, R:100%, F: 71.8% Diabetes Forum (with RSC) P: 86.5%, R:86.4%, F:86.4% (without RSC) P: 68.5%, R:100%, F: 81.3% MedHelp (heart disease) (with RSC) P: 89.6%, R:91.4%, F:90.5% (without RSC) P: 63.5%, R:100%, F: 77.7% |
| ***Retrieving relevant information based on user query (n=1)*** | | |
| Cameron 2014[13] | **Query matcher:** to retrieve and filter the relevant documents for a given user query, based on query interpretation and document annotations in the text data. | None provided |

**Abbreviations:** P - Precision; R - Recall; F - F-measure; A - Accuracy; AUC - Area under the curve

# Appendix 17. Utility and challenges of social media data for pharmacovigilance

| **Article ID** | **Overall Utility** | **Overall Challenges** |
| --- | --- | --- |
| Abou Taam 2014[7] | Captures personal and social perceptions and consequences of treatment and adverse events; Useful for risk communication. | NA |
| Akay 2015[8] | Captures personal and social perceptions and consequences of treatment and adverse events; promising early warning system. | Non-standard corpus (language, format, content); difficult to draw complex semantic relationships from corpus. |
| Alvaro 2015[9] | Expansive data source. | NA |
| Benton 2011[10] | Promising early warning system; able to discover undocumented or rare adverse events. | Inadequate information to draw causality. |
| Beusterien 2013[11] | Captures personal and social perceptions and consequences of treatment and adverse events; useful for risk communication; complementary to traditional post-marketing safety surveillance. | Inadequate information to draw causality; lacks comprehensive medical and demographic information; may lack representative population. |
| Bian 2012[12] | Complementary to traditional post-marketing safety surveillance; promising early warning system. | Noise in signal detection; non-standard corpus (language, format, content). |
| Cameron 2014[13] | NA | NA |
| Carbonell 2015[14] | Expansive data source; complementary to traditional post-marketing safety surveillance; able to identify undocumented drug interactions; Captures personal and social perceptions and consequences of treatment and adverse events. | Difficult to draw complex semantic relationships from corpus. |
| Chary 2014[15] | Expansive data source; computationally efficient; not biased to serious adverse events; captures prescription drug misuse/abuse; complementary to traditional post-marketing safety surveillance. | May lack representative population; Lacks comprehensive medical and demographic information; difficult to draw complex semantic relationships from corpus. |
| Chee 2009[16] | Complementary to traditional post-marketing safety surveillance; hypothesis-generating. | Noise in signal detection; inadequate information to draw causality. |
| Chee 2011[17] | Complementary to traditional post-marketing safety surveillance; promising early warning system; not biased to serious adverse events. | Resource-intensive to train machine on big data; difficult to draw complex semantic relationships from corpus; lacks comprehensive medical and demographic information; not a balanced coverage of all drugs and medical conditions; non-standard corpus (language, format, content). |
| Coloma 2015[18] | Captures personal and social perceptions and consequences of treatment and adverse events; complementary to traditional post-marketing safety surveillance. | May lack representative population. |
| Correia 2016[19] | Expansive data source; complementary to traditional post-marketing safety surveillance. | May lack representative population; non-standard corpus (language, format, content). |
| Elhadad 2014[20] | NA | NA |
| Freifeld 2014a[21] | Expansive data source; complementary to traditional post-marketing safety surveillance; Hypothesis-generating. | Non-standard corpus (language, format, content); noise in signal detection. |
| Freifeld 2014b[22] | Complementary to traditional post-marketing safety surveillance. | May lack representative population; not a balanced coverage of all drugs and medical conditions; difficult to draw complex semantic relationships from corpus; subjective, incomplete or misinformation; lacks comprehensive medical and demographic information. |
| Ginn 2014[23, 81] | Able to extract complex medical concepts. | Data acquisition challenges due to host site restrictions; non-standard corpus (language, format, content); not a balanced coverage of all drugs and medical conditions. |
| Gupta 2014[24] | Captures personal and social perceptions and consequences of treatment and adverse events. | Difficult to draw complex semantic relationships from corpus. |
| Hadzi-Puric 2012[25] | Able to discover undocumented or rare adverse events. | Non-standard corpus (language, format, content); lacks comprehensive medical and demographic information. |
| Hanson 2013a[26] | Findings are comparable to traditional systems. | Non-standard corpus (language, format, content); noise in signal detection. |
| Hanson 2013b[27] | NA | NA |
| Hughes 2011[28] | NA | NA |
| Jimeno-Yepes 2015[29] | NA | NA |
| Johnson 2013[30] | NA | Inadequate information to draw causality; non-standard corpus (language, format, content). |
| Karimi 2015b[32] | Able to discover undocumented or rare adverse events. | Non-standard corpus (language, format, content). |
| Karimi 2015a[31, 77] | NA | Non-standard corpus (language, format, content); difficult to draw complex semantic relationships from corpus; noise in signal detection; subjective, incomplete or misinformation. |
| Kmetz 2011[33] | NA | NA |
| Leaman 2010[34] | Complementary to traditional post-marketing safety surveillance; promising early warning system; not biased to serious adverse events. | Lacks comprehensive medical and demographic information. |
| Liu 2011[35, 78] | Complementary to traditional post-marketing safety surveillance; able to discover undocumented or rare adverse events. | Non-standard corpus (language, format, content). |
| Liu 2014[36] | Complementary to traditional post-marketing safety surveillance; not biased to severe adverse events; Able to identify undocumented drug interactions. | NA |
| Liu 2015a[37, 79, 80] | Complementary to traditional post-marketing safety surveillance; not biased to severe adverse events; captures personal and social perceptions and consequences of treatment and adverse events. | Difficult to draw complex semantic relationships from corpus. |
| Liu 2015b[38] | Able to discover undocumented or rare adverse events; computationally efficient. | Not a balanced coverage of all drugs and medical conditions. |
| Mao 2013[39] | Complementary to traditional post-marketing safety surveillance; wide geographic capture; Information of adherence related to adverse events. | Duplication of data (double-counting). |
| McGinley 2015[40] | Wide geographic capture. | Non-standard corpus (language, format, content). |
| Medawar 2002[41] | Complementary to traditional post-marketing safety surveillance; captures personal and social perceptions and consequences of treatment and adverse events. | NA |
| Metke-Jimenez 2014[42] | Complementary to traditional post-marketing safety surveillance. | Noise in signal detection; non-standard corpus (language, format, content); Inadequate information to draw causality. |
| Metke-Jimenez 2015[43] | Complementary to traditional post-marketing safety surveillance. | Non-standard corpus (language, format, content). |
| Nadarajah 2015[44] | Computationally efficient; expansive data source. | NA |
| Nikfarjam 2011[45] | NA | Non-standard corpus (language, format, content). |
| Nikfarjam 2015[46] | Able to extract complex medical concepts. | Non-standard corpus (language, format, content). |
| Oleson 2013[47] | Expansive data source; not biased to severe adverse events. | NA |
| Pages 2014[48] | Complementary to traditional post-marketing safety surveillance; not biased to severe adverse events. | Inadequate information to draw causality. |
| Patki 2014[49] | NA | Not a balanced coverage of all drugs and medical conditions; Noise in signal detection. |
| Pimpalkhute 2014[50] | NA | NA |
| Powell 2016[51] | Complementary to traditional post-marketing safety surveillance; captures personal and social perceptions and consequences of treatment and adverse events. | Data acquisition challenges due to host site restrictions; non-standard corpus (language, format, content); processing multi-lingual corpus. |
| Risson 2016[52] | Expansive data source; captures personal and social perceptions and consequences of treatment and adverse events. | Non-standard corpus (language, format, content); duplication of data (double-counting); lacks comprehensive medical and demographic information; may lack representative population; difficult to draw complex semantic relationships from corpus. |
| Rizo 2011[53] | Complementary to traditional post-marketing safety surveillance; computationally efficient; promising early warning system. | Non-standard corpus (language, format, content); data acquisition challenges due to host site restrictions; may lack representative population. |
| Sampathkumar 2014[54] | Promising early warning system; able to discover undocumented or rare adverse events; complementary to traditional post-marketing safety surveillance. | Non-standard corpus (language, format, content); subjective incomplete or misinformation. |
| Sarker 2015[55] | Expansive data source. | Non-standard corpus (language, format, content); difficult to draw complex semantic relationships from corpus. |
| Sarker 2016a[56] | Expansive data source. | Subjective, incomplete or misinformation; duplication of data (double-counting). |
| Sarker 2016b[57] | Captures prescription drug misuse/abuse. | Noise in signal detection; subjective incomplete or misinformation; non-standard corpus (language, format, content); may lack representative population. |
| Sarrazin 2014[58] | Captures personal and social perceptions and consequences of treatment and adverse events; complementary to traditional post-marketing safety surveillance; able to discover undocumented or rare adverse events. | May lack representative population. |
| Scanfeld 2010[59] | Captures prescription drug misuse/abuse. | May lack representative population; subjective incomplete or misinformation. |
| Schroder 2007[60] | Captures personal and social perceptions and consequences of treatment and adverse events; computationally efficient; complementary to traditional post-marketing safety surveillance; able to discover undocumented or rare adverse events. | May lack representative population. |
| Segura-Bedmar 2014[61, 82] | Captures personal and social perceptions and consequences of treatment and adverse events. | Processing multi-lingual corpus. |
| Segura-Bedmar 2015[62] | NA | Non-standard corpus (language, format, content); noise in signal detection; difficult to draw complex semantic relationships from corpus. |
| Shutler 2015[63] | Captures prescription drug misuse/abuse; useful for risk communication. | Non-standard corpus (language, format, content); difficult to draw complex semantic relationships from corpus. |
| Topaz 2016[64] | Able to discover undocumented or rare adverse events; complementary to traditional post-marketing safety surveillance. | Inadequate information to draw causality. |
| White 2013[65] | Complementary to traditional post-marketing safety surveillance. | Non-standard corpus (language, format, content). |
| White 2014[66] | Complementary to traditional post-marketing safety surveillance; can be more accurate than spontaneous reporting systems; expansive data source. | Noise in signal detection; inadequate information to draw causality. |
| Whitman 2014[67] | Captures personal and social perceptions and consequences of treatment and adverse events. | Duplication of data (double-counting); may lack representative population; lacks comprehensive medical and demographic information. |
| Wu 2013[68] | Complementary to traditional post-marketing safety surveillance; able to discover undocumented or rare adverse events. | Difficult to draw complex semantic relationships from corpus. |
| Yang 2012a[69] | Promising early warning system; can be more accurate than spontaneous reporting systems; expansive data source. | Non-standard corpus (language, format, content). |
| Yang 2012b[70] | Complementary to traditional post-marketing safety surveillance. | Non-standard corpus (language, format, content); difficult to draw complex semantic relationships from corpus. |
| Yang 2013[71] | Expansive data source. | Non-standard corpus (language, format, content). |
| Yang 2015a[72] | Promising early warning system; computationally efficient. | Non-standard corpus (language, format, content). |
| Yang 2015b[73] | Complementary to traditional post-marketing safety surveillance; promising early warning system. | Resource-intensive to train machine on big data. |
| Yates 2013a[74] | NA | NA |
| Yates 2013b[75] | Able to discover undocumented or rare adverse events. | NA |
| Yom-Tov 2013[76] | Computationally efficient; wide geographic capture; complementary to traditional post-marketing safety surveillance; expansive data source. | Noise in signal detection; inadequate information to draw causality; may lack representative population; processing multi-lingual corpus. |

**Abbreviations:** NA – Not applicable

# References

1. **Health Canada. Biologics, Radiopharmaceuticals and Genetic Therapies. 2016.** [**http://www.hc-sc.gc.ca/dhp-mps/brgtherap/index-eng.php**](http://www.hc-sc.gc.ca/dhp-mps/brgtherap/index-eng.php).

2. **Health Canada. Drug and Health Products. 2017.** [**http://www.hc-sc.gc.ca/dhp-mps/pubs/drug-medic/index-eng.php**](http://www.hc-sc.gc.ca/dhp-mps/pubs/drug-medic/index-eng.php)**.**

3. **Health Canada. Medical Devices. 2012.** [**http://www.hc-sc.gc.ca/dhp-mps/md-im/index-eng.php**](http://www.hc-sc.gc.ca/dhp-mps/md-im/index-eng.php).

4. **Health Canada. Natural and Non-prescription Health Products. 2016.** [**http://www.hc-sc.gc.ca/dhp-mps/prodnatur/index-eng.php**](http://www.hc-sc.gc.ca/dhp-mps/prodnatur/index-eng.php).

5. **WSIB Ontario. Programs. 2017.** [**http://www.wsib.on.ca/WSIBPortal/faces/WSIBArticlePage?fGUID=835502100635000404&_afrLoop=916070698082000&_afrWindowMode=0&_afrWindowId=null#%40%3F_afrWindowId%3Dnull%26_afrLoop%3D916070698082000%26_afrWindowMode%3D0%26fGUID%3D835502100635000404%26_adf.ctrl-state%3D12han0k5em_4**](http://www.wsib.on.ca/WSIBPortal/faces/WSIBArticlePage?fGUID=835502100635000404&_afrLoop=916070698082000&_afrWindowMode=0&_afrWindowId=null#%40%3F_afrWindowId%3Dnull%26_afrLoop%3D916070698082000%26_afrWindowMode%3D0%26fGUID%3D835502100635000404%26_adf.ctrl-state%3D12han0k5em_4)**.**

6. **Health Services** [<http://www.who.int/topics/health_services/en/>]

7. Abou Taam M, Rossard C, Cantaloube L, Bouscaren N, Pochard L, Montastruc F, Montastruc J, Bagheri H: **Analyze of internet narratives on patient websites before and after benfluorex withdrawal and media coverage**. *Fundam Clin Pharmacol* 2012, **26**:79-80.

8. Akay A, Dragomir A, Erlandsson B-E: **Network-based modeling and intelligent data mining of social media for improving care**. *IEEE journal of biomedical and health informatics* 2015, **19**(1):210-218.

9. Alvaro N, Conway M, Doan S, Lofi C, Overington J, Collier N: **Crowdsourcing Twitter annotations to identify first-hand experiences of prescription drug use**. *Journal of biomedical informatics* 2015, **58**:280-287.

10. Benton A, Ungar L, Hill S, Hennessy S, Mao J, Chung A, Leonard CE, Holmes JH: **Identifying potential adverse effects using the web: A new approach to medical hypothesis generation**. *Journal of biomedical informatics* 2011, **44**(6):989-996.

11. Beusterien K, Tsay S, Gholizadeh S, Su Y: **Real-world experience with colorectal cancer chemotherapies: patient web forum analysis**. *Ecancermedicalscience* 2013, **7**:361.

12. Bian J, Topaloglu U, Yu F: **Towards large-scale twitter mining for drug-related adverse events**. In: *Proceedings of the 2012 international workshop on Smart health and wellbeing: 2012*: ACM; 2012: 25-32.

13. Cameron D, Sheth AP, Jaykumar N, Thirunarayan K, Anand G, Smith GA: **A hybrid approach to finding relevant social media content for complex domain specific information needs**. *Web Semantics: Science, Services and Agents on the World Wide Web* 2014, **29**:39-52.

14. Carbonell P, Mayer MA, Bravo À: **Exploring brand-name drug mentions on Twitter for pharmacovigilance**. *Stud Health Technol Inform* 2015, **210**:55-59.

15. Chary M, Park EH, McKenzie A, Sun J, Manini AF, Genes N: **Signs & symptoms of dextromethorphan exposure from YouTube**. *PLoS One* 2014, **9**(2):e82452.

16. Chee BW, Berlin R, Schatz BR: **Measuring population health using personal health messages**. In: *AMIA: 2009*; 2009.

17. Chee BW, Berlin R, Schatz B: **Predicting adverse drug events from personal health messages**. In: *AMIA Annu Symp Proc: 2011*; 2011: 217-226.

18. Coloma PM, Becker B, Sturkenboom MC, van Mulligen EM, Kors JA: **What Can Social Media Networks Contribute To Medicines Safety Surveillance?** In: *Pharmacoepidemiol Drug Saf: 2015*: Wiley-Blackwell 2015: 467-468.

19. Correia RB, Li L, Rocha LM: **Monitoring potential drug interactions and reactions via network analysis of instagram user timelines**. In: *Pacific Symposium on Biocomputing Pacific Symposium on Biocomputing: 2016*: NIH Public Access; 2016: 492.

20. Elhadad N, Zhang S, Driscoll P, Brody S: **Characterizing the sublanguage of online breast cancer forums for medications, symptoms, and emotions**. In: *Proc AMIA Annual Fall Symposium: 2014*; 2014.

21. Freifeld CC, Brownstein JS, Menone CM, Bao W, Filice R, Kass-Hout T, Dasgupta N: **Digital drug safety surveillance: monitoring pharmaceutical products in twitter**. *Drug Saf* 2014, **37**(5):343-350.

22. Freifeld CC: **Digital Pharmacovigilance: the medwatcher system for monitoring adverse events through automated processing of internet social media and crowdsourcing**. 2014.

23. Ginn R, Pimpalkhute P, Nikfarjam A, Patki A, O’Connor K, Sarker A, Smith K, Gonzalez G: **Mining Twitter for adverse drug reaction mentions: a corpus and classification benchmark**. In: *Proceedings of the fourth workshop on building and evaluating resources for health and biomedical text processing: 2014*: Citeseer; 2014.

24. Gupta S, MacLean DL, Heer J, Manning CD: **Induced lexico-syntactic patterns improve information extraction from online medical forums**. *J Am Med Inform Assoc* 2014, **21**(5):902-909.

25. Hadzi-Puric J, Grmusa J: **Automatic drug adverse reaction discovery from parenting websites using disproportionality methods**. In: *Proceedings of the 2012 International Conference on Advances in Social Networks Analysis and Mining (ASONAM 2012): 2012*: IEEE Computer Society; 2012: 792-797.

26. Hanson CL, Burton SH, Giraud-Carrier C, West JH, Barnes MD, Hansen B: **Tweaking and tweeting: exploring Twitter for nonmedical use of a psychostimulant drug (Adderall) among college students**. *J Med Internet Res* 2013, **15**(4):e62.

27. Hanson CL, Cannon B, Burton S, Giraud-Carrier C: **An exploration of social circles and prescription drug abuse through Twitter**. *J Med Internet Res* 2013, **15**(9):e189.

28. Hughes S, Cohen D: **Can online consumers contribute to drug knowledge? A mixed-methods comparison of consumer-generated and professionally controlled psychotropic medication information on the internet**. *J Med Internet Res* 2011, **13**(3):e53.

29. Jimeno-Yepes A, MacKinlay A, Han B, Chen Q: **Identifying Diseases, Drugs, and Symptoms in Twitter**. *Stud Health Technol Inform* 2014, **216**:643-647.

30. Johnson HK, Nancy: **A side effect of social media. What can twitter tell us about adverse drug reactions**. In: *UKMI 39th Professional Development Seminar: 2013*; 2013.

31. Karimi S, Metke-Jimenez A, Kemp M, Wang C: **Cadec: A corpus of adverse drug event annotations**. *Journal of biomedical informatics* 2015, **55**:73-81.

32. Karimi S, Metke-Jimenez A, Nguyen A: **CADEminer: A System for Mining Consumer Reports on Adverse Drug Side Effects**. In: *Proceedings of the Eighth Workshop on Exploiting Semantic Annotations in Information Retrieval: 2015*: ACM; 2015: 47-50.

33. Kmetz J: **Pharmaceutical industry special report: Adverse Event Reporting in Social Media**. In*.*: Visible; 2011.

34. Leaman R, Wojtulewicz L, Sullivan R, Skariah A, Yang J, Gonzalez G: **Towards internet-age pharmacovigilance: extracting adverse drug reactions from user posts to health-related social networks**. In: *Proceedings of the 2010 workshop on biomedical natural language processing: 2010*: Association for Computational Linguistics; 2010: 117-125.

35. Liu J, Li A, Seneff S: **Automatic drug side effect discovery from online patient-submitted reviews: Focus on statin drugs**. In: *Proceedings of First International Conference on Advances in Information Mining and Management (IMMM): 2011; Barcelona, Spain*; 2011: 23-29.

36. Liu X, Liu J, Chen H: **Identifying adverse drug events from health social media: a case study on heart disease discussion forums**. In: *International Conference on Smart Health: 2014*: Springer; 2014: 25-36.

37. Liu X, Chen H: **A research framework for pharmacovigilance in health social media: Identification and evaluation of patient adverse drug event reports**. *Journal of biomedical informatics* 2015, **58**:268-279.

38. Liu X, Chen H: **Identifying Adverse Drug Events from Health Social Media Using Distant Supervision**. In: *INFORMS Conference on Information Systens and Technology.* Philadelphia; 2015.

39. Mao JJ, Chung A, Benton A, Hill S, Ungar L, Leonard CE, Hennessy S, Holmes JH: **Online discussion of drug side effects and discontinuation among breast cancer survivors**. *Pharmacoepidemiol Drug Saf* 2013, **22**(3):256-262.

40. McGinley M, Alinia H, Kuo S, Huang KE, Feldman SR: **Patient perspectives on low level light therapy and laser therapies for rosacea-associated persistent facial redness**. *Dermatol Online J* 2015, **21**(2).

41. Medawar C, Herxheimer A, Bell A, Jofre S: **Paroxetine, Panorama and user reporting of ADRs: Consumer intelligence matters in clinical practice and post‐marketing drug surveillance**. *Int J Risk Saf Med* 2002, **15**(3, 4):161-169.

42. Metke-Jimenez A, Karimi S, Paris C: **Evaluation of text-processing algorithms for adverse drug event extraction from social media**. In: *Proceedings of the first international workshop on Social media retrieval and analysis: 2014*: ACM; 2014: 15-20.

43. Metke-Jimenez A, Karimi S: **Concept extraction to identify adverse drug reactions in medical forums: A comparison of algorithms**. *arXiv preprint arXiv:150406936* 2015.

44. Nadarajah S: **Monitoring Adverse Events in Pharma’s Patient Support Programs**. In*.*: IMS Health; 2015.

45. Nikfarjam A, Gonzalez GH: **Pattern mining for extraction of mentions of adverse drug reactions from user comments**. In: *AMIA Annu Symp Proc: 2011*; 2011: 1019-1026.

46. Nikfarjam A, Sarker A, O’Connor K, Ginn R, Gonzalez G: **Pharmacovigilance from social media: mining adverse drug reaction mentions using sequence labeling with word embedding cluster features**. *J Am Med Inform Assoc* 2015, **22**(3):671-681.

47. **Discovering Drug Side Effects with Crowdsourcing** [<https://www.crowdflower.com/discovering-drug-side-effects-with-crowedsourcing/>]

48. Pages A, Bondon-Guitton E, Montastruc JL, Bagheri H: **Undesirable effects related to oral antineoplastic drugs: comparison between patients’ internet narratives and a national pharmacovigilance database**. *Drug Saf* 2014, **37**(8):629-637.

49. Patki A, Sarker A, Pimpalkhute P, Nikfarjam A, Ginn R, O’Connor K, Smith K, Gonzalez G: **Mining adverse drug reaction signals from social media: going beyond extraction**. *Proceedings of BioLinkSig* 2014, **2014**:1-8.

50. Pimpalkhute P, Patki A: **Phonetic spelling filter for keyword selection in drug mention mining from social media**. 2014.

51. Powell GE, Seifert HA, Reblin T, Burstein PJ, Blowers J, Menius JA, Painter JL, Thomas M, Pierce CE, Rodriguez HW: **Social media listening for routine post-marketing safety surveillance**. *Drug Saf* 2016, **39**(5):443-454.

52. Risson V, Saini D, Bonzani I, Huisman A, Olson M: **Validation of Social Media Analysis for Outcomes Research: Identification of Drivers of Switches between oral and Injectable Therapies for Multiple Sclerosis**. *Value Health* 2015, **18**(7):A729.

53. Rizo C, Deshpande A, Ing A, Seeman N: **A rapid, Web-based method for obtaining patient views on effects and side-effects of antidepressants**. *J Affect Disord* 2011, **130**(1):290-293.

54. Sampathkumar H, Chen X-w, Luo B: **Mining adverse drug reactions from online healthcare forums using hidden Markov model**. *BMC Med Inform Decis Mak* 2014, **14**(1):1.

55. Sarker A, Gonzalez G: **Portable automatic text classification for adverse drug reaction detection via multi-corpus training**. *Journal of biomedical informatics* 2015, **53**:196-207.

56. Sarker A, Nikfarjam A, Gonzalez G: **Social media mining shared task workshop**. In: *Proceedings of the Pacific Symposium on Biocomputing: 2016*; 2016.

57. Sarker A, O’Connor K, Ginn R, Scotch M, Smith K, Malone D, Gonzalez G: **Social media mining for toxicovigilance: automatic monitoring of prescription medication abuse from Twitter**. *Drug Saf* 2016, **39**(3):231-240.

58. Sarrazin MSV, Cram P, Mazur A, Ward M, Reisinger HS: **Patient perspectives of dabigatran: analysis of online discussion forums**. *The Patient-Patient-Centered Outcomes Research* 2014, **7**(1):47-54.

59. Scanfeld D, Scanfeld V, Larson EL: **Dissemination of health information through social networks: Twitter and antibiotics**. *Am J Infect Control* 2010, **38**(3):182-188.

60. Schröder S, Zöllner YF, Schaefer M: **Drug related problems with Antiparkinsonian agents: consumer Internet reports versus published data**. *Pharmacoepidemiol Drug Saf* 2007, **16**(10):1161-1166.

61. Segura-Bedmar I, Revert R, Martínez P: **Detecting drugs and adverse events from Spanish health social media streams**. In: *Proceedings of the 5th International Workshop on Health Text Mining and Information Analysis (Louhi)@ EACL: 2014*; 2014: 106-115.

62. Segura-Bedmar I, Martínez P, Revert R, Moreno-Schneider J: **Exploring Spanish health social media for detecting drug effects**. *BMC Med Inform Decis Mak* 2015, **15**(2):1.

63. Shutler L, Nelson LS, Portelli I, Blachford C, Perrone J: **Drug use in the Twittersphere: a qualitative contextual analysis of tweets about prescription drugs**. *J Addict Dis* 2015, **34**(4):303-310.

64. Topaz M, Lai K, Dhopeshwarkar N, Seger DL, Sa’adon R, Goss F, Rozenblum R, Zhou L: **Clinicians’ Reports in Electronic Health Records Versus Patients’ Concerns in Social Media: A Pilot Study of Adverse Drug Reactions of Aspirin and Atorvastatin**. *Drug Saf* 2016, **39**(3):241-250.

65. White RW, Tatonetti NP, Shah NH, Altman RB, Horvitz E: **Web-scale pharmacovigilance: listening to signals from the crowd**. *J Am Med Inform Assoc* 2013, **20**(3):404-408.

66. White RW, Harpaz R, Shah NH, DuMouchel W, Horvitz E: **Toward enhanced pharmacovigilance using patient-generated data on the internet**. *Clin Pharmacol Ther* 2014, **96**(2):239.

67. Whitman CB, Reid MW, Arnold C, Patel H, Ursos L, Sa'adon R, Pourmorady J, Spiegel B: **Balancing opioid-induced gastrointestinal side effects with pain management: Insights from the online community**. *Journal of opioid management* 2014, **11**(5):383-391.

68. Wu H, Fang H, Stanhope S: **Exploiting online discussions to discover unrecognized drug side effects**. *Methods Inf Med* 2013, **52**(2):152-159.

69. Yang CC, Jiang L, Yang H, Tang X: **Detecting signals of adverse drug reactions from health consumer contributed content in social media**. In: *Proceedings of ACM SIGKDD Workshop on Health Informatics: 2012*; 2012.

70. Yang CC, Yang H, Jiang L, Zhang M: **Social media mining for drug safety signal detection**. In: *Proceedings of the 2012 international workshop on Smart health and wellbeing: 2012*: ACM; 2012: 33-40.

71. Yang H, Yang CC: **Harnessing social media for drug-drug interactions detection**. In: *Healthcare Informatics (ICHI), 2013 IEEE International Conference on: 2013*: IEEE; 2013: 22-29.

72. Yang M, Kiang M, Shang W: **Filtering big data from social media–Building an early warning system for adverse drug reactions**. *Journal of biomedical informatics* 2015, **54**:230-240.

73. Yang CC, Yang H: **Exploiting Social Media with Tensor Decomposition for Pharmacovigilance**. In: *2015 IEEE International Conference on Data Mining Workshop (ICDMW): 2015*: IEEE; 2015: 188-195.

74. Yates A, Goharian N, Frieder O: **Extracting adverse drug reactions from forum posts and linking them to drugs**. In: *Proceedings of the 2013 ACM SIGIR Workshop on Health Search and Discovery: 2013*; 2013.

75. Yates A, Goharian N: **ADRTrace: Detecting Expected and Unexpected Adverse Drug Reactions from User Reviews on Social Media Sites**. In: *Advances in Information Retrieval: 35th European Conference on IR Research.* Edited by Serdyukov P, Braslavski P, Kuznetsov SO, Kamps J, Rüger S, Agichtein E, Segalovich I, Yilmaz E. Berlin, Heidelberg: Springer Berlin Heidelberg; 2013: 816-819.

76. Yom-Tov E, Gabrilovich E: **Postmarket drug surveillance without trial costs: discovery of adverse drug reactions through large-scale analysis of web search queries**. *J Med Internet Res* 2013, **15**(6):e124.

77. Karimi S, Kim S, Cavedon L: **Drug side-effects: What do patient forums reveal**. In: *The second international workshop on Web science and information exchange in the medical Web: 2011*: ACM; 2011: 10-11.

78. Li YA: **Medical data mining: Improving information accessibility using online patient drug reviews**. Massachusetts Institute of Technology; 2011.

79. Liu X, Chen H: **AZDrugMiner: an information extraction system for mining patient-reported adverse drug events in online patient forums**. In: *International Conference on Smart Health: 2013*: Springer; 2013: 134-150.

80. Liu X, Chen H: **Identifying adverse drug events from patient social media: A case study for diabetes**. *IEEE Intelligent Systems* 2015, **30**(3):44-51.

81. O’Connor K, Pimpalkhute P, Nikfarjam A, Ginn R, Smith KL, Gonzalez G: **Pharmacovigilance on Twitter? Mining Tweets for adverse drug reactions**. In: *AMIA Annual Symposium Proceedings: 2014*: American Medical Informatics Association; 2014: 924.

82. Segura-Bedmar I, De La Peña S, Martınez P: **Extracting drug indications and adverse drug reactions from Spanish health social media**. In: *Proceedings of BioNLP: 2014*; 2014: 98-106.

83. Wang C, Karimi S: **Differences between social media and regulatory databases in adverse drug reaction discovery**. In: *Proceedings of the first international workshop on Social media retrieval and analysis: 2014*: ACM; 2014: 13-14.
